# *Isospora* and *Lankesterella* Parasites (Eimeriidae, Apicomplexa) of Passeriform Birds in Europe: Infection Rates, Phylogeny, and Pathogenicity

**DOI:** 10.3390/pathogens13040337

**Published:** 2024-04-18

**Authors:** Carina Keckeisen, Alžbeta Šujanová, Tanja Himmel, Julia Matt, Nora Nedorost, Carolina R. F. Chagas, Herbert Weissenböck, Josef Harl

**Affiliations:** 1 Institute of Pathology, Department of Biological Sciences and Pathobiology, University of Veterinary Medicine Vienna, 1210 Vienna, Austria; carina.keckeisen@vetmeduni.ac.at (C.K.); tanja.himmel@vetmeduni.ac.at (T.H.);; 2 Institute of Zoology, Slovak Academy of Sciences, 845 06 Bratislava, Slovakia; alzbeta.sujanova@savba.sk; 3 Nature Research Centre, 084 12 Vilnius, Lithuania; carolina.chagas@gamtc.lt; 4 Clinical Institutes of the MedUni Vienna, Department of Pathology, Medical University of Vienna, 1090 Vienna, Austria

**Keywords:** *Isospora*, *Lankesterella*, tissue stages, wild birds, chromogenic in situ hybridization, *cytochrome b*, *cytochrome c oxidase I*

## Abstract

Wild birds are common hosts to numerous intracellular parasites such as single-celled eukaryotes of the family Eimeriidae (order Eucoccidiorida, phylum Apicomplexa). We investigated the infection rates, phylogeny, and pathogenicity of *Isospora* and *Lankesterella* parasites in wild and captive passerine birds. Blood and tissue samples of 815 wild and 15 deceased captive birds from Europe were tested using polymerase chain reaction and partial sequencing of the mitochondrial *cytochrome b* and *cytochrome c oxidase I* and the nuclear *18S* rRNA gene. The infection rate for *Lankesterella* in wild birds was 10.7% compared to 5.8% for *Isospora*. Chromogenic in situ hybridization with probes targeting the parasites’ *18S* rRNA was employed to identify the parasites’ presence in multiple organs, and hematoxylin–eosin staining was performed to visualize the parasite stages and assess associated lesions. *Isospora* parasites were mainly identified in the intestine, spleen, and liver. Extraintestinal tissue stages of *Isospora* were accompanied by predominantly lymphohistiocytic inflammation of varying severity. *Lankesterella* was most frequently detected in the spleen, lung, and brain; however, infected birds presented only a low parasite burden without associated pathological changes. These findings contribute to our understanding of *Isospora* and *Lankesterella* parasites in wild birds.

## 1. Introduction

Apicomplexa is a diverse group of single-celled eukaryotic protozoans, many of which are obligate endoparasites of animals. The most common and studied apicomplexan parasites of birds belong to the orders Haemosporida (class Aconoidasida) and Eucoccidiorida (class Conoidasida). Parasites of the genera *Isospora* and *Lankesterella* (family Eimeriidae, order Eucoccidiorida) have received less attention, and the effects of their infections in wild birds are less understood. Additionally, these two parasite genera were often confused because their life cycles remain insufficiently investigated to this day, and their life stages in host tissue closely resemble each other [1,2,3,4,5]. Several of the latter studies have described the life stages of *Lankesterella* in bird tissues, but based on the currently available information, these observations likely referred to *Isospora* [6]. It is also necessary to mention that several parasites described as *Hepatozoon* species probably belong to *Lankesterella*, which has been pointed out previously [7,8,9].

**Figure 1:**
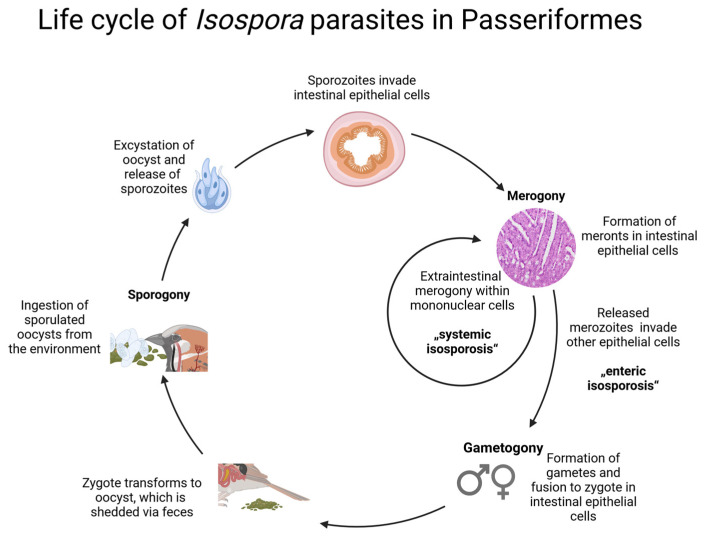
Schematic illustration of the life cycle of *Isospora* parasites in Passeriformes based on descriptions in [13,17,18]. The life cycle of *Isospora* in avian hosts consists of an endogenous and exogenous part whereby transmission occurs by the ingestion of infectious sporulated oocysts (tetrasporozoic diplosporocystic) from the environment via the fecal–oral route. Excystation takes place in the bird’s intestine, releasing sporozoites that invade epithelial cells of the mucosa. Subsequently, merogony occurs, producing merozoites which invade other epithelial cells and undergo replication. Gametogony is initiated by the merozoites from the last merogony generation. Micro- and macrogamonts fuse during fertilization to form a zygote (enteric isosporosis). Unsporulated oocysts are excreted with the feces to sporulate in the environment within a few days to become infectious for the next avian host. In systemic isosporosis, formerly known as atoxoplasmosis, merozoites produced in intestinal epithelial cells invade mononuclear cells and leave the intestine, leading to extraintestinal merogony.

The genus *Isospora* Schneider, 1881, is currently assumed to comprise a group of monoxenous coccidian parasites of passeriform birds [10], including parasites previously assigned to the genus *Atoxoplasma* Garnham, 1950, which is a synonym of *Isospora* according to Barta et al. [11]. Transmission of *Isospora* between vertebrate hosts occurs via the shedding of sporulated oocysts through feces and oral intake by other individuals [12] (Figure 1). The parasites infect the intestines and can spread to other organs via extraintestinal merozoites in blood-circulating mononuclear cells. Commonly affected organs are the spleen, liver, heart, and lung [10,12]. *Isospora* species in passerine birds encompass organisms with short intestinal life cycles (enteric isosporosis) and others with extended life cycles, including extraintestinal merogony (systemic isosporosis) [12,13]. Historically, there were suggestions that intestinal and extraintestinal forms represent distinct parasites [14]; however, molecular studies of these parasites from various tissues revealed that these forms belong to the same parasite species [12,15,16].

Isosporosis can be subclinical or lead to severe disease with a fatal outcome [13,17]. While enteric isosporosis affects birds from diverse orders, systemic isosporosis has evolved only in birds of the order Passeriformes according to Flach et al. [13]. Clinical signs of infection are non-specific and may include apathy, loss of appetite, diarrhea, dehydration, progressive weight loss, reduction in pectoral musculature, dyspnea, tachypnea, ruffled feathering, abdominal distension, retraced neck, and loss of balance [18,19,20,21,22]. Because these symptoms often go unnoticed, detection of disease *ante mortem* is very challenging, especially in wild birds. High mortality associated with virulent systemic infection was predominantly observed in fledglings but may also occur in adults [18,23]. Traditionally, *Isospora* parasites are identified from feces rather than blood, and currently known species are distinguished based on their oocyst morphology. Only a few studies have investigated *Isospora* parasites from birds using molecular techniques [13,24,25,26,27]. 

**Figure 2:**
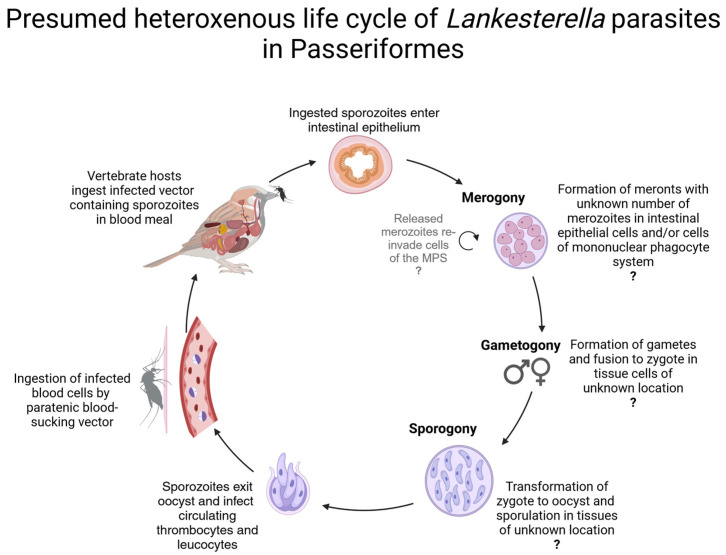
Schematic illustration of the proposed heteroxenous life cycle of *Lankesterella* parasites in Passeriformes based on descriptions in published studies on birds [6] and amphibians [31]. Transmission of *Lankesterella* parasites in birds presumably occurs by predation of infected invertebrate vectors, followed by merogony, gametogony, and sporogony, likely in intestinal epithelial cells and/or cells of the mononuclear phagocytic system, producing sporozoites which invade leucocytes and thrombocytes. Infected circulating blood cells are then ingested by hematophagous vectors without further development. After their uptake by the vectors, the parasites’ sporozoites can persist within them for a long time and were seen up to 42 days post-infection in *Aedes aegypti*. In this illustration, a mosquito is shown as a potential vector, but it is still not known which vectors are actually capable of transmitting *Lankesterella*.

The genus *Lankesterella* Labbé, 1899, was first described from amphibians [28]. The parasites are assumed to follow a heteroxenous life cycle with merogony, gametogony, and development of sporulated oocysts of *Lankesterella* taking place in cells of the mononuclear phagocyte system throughout the anuran body, followed by excystation to release sporozoites which invade erythrocytes [29,30,31,32,33,34]. Blood-sucking invertebrate vectors such as leeches subsequently ingest the blood-circulating sporozoites and transmit them to another host during their next blood meal [29,30,31]. An experimental infection of *Bufo marinus* has also been reported in which transmission occurred through the forced oral ingestion of liver tissue and blood from infected toads [32]. In recent years, studies have also been published on *Lankesterella* species in reptiles [35,36,37,38]. Transmission occurs in saurian hosts by predation on the infected invertebrate vectors such as dipterans and acarines [37,39], and the parasites are considered to be highly host-specific [38]. As in anurans, merogony, gametogony, and sporogony of *Lankesterella* presumably take place in the saurian host, with invasive sporozoites developing as blood stages that infect erythrocytes as well as leucocytes [36]. During the vector’s blood meal, sporozoites are ingested to complete the life cycle, whereby no further development takes place in the vector serving as a paratenic host [35]. Research on avian *Lankesterella* parasites is a relatively young field [6,13,40]. It is likely that the *Lankesterella* life cycle is similar to the one described for amphibians, with merogony, gametogony, and sporogony of *Lankesterella* parasites taking place entirely within the vertebrate host, leading to the development of sporozoites, which infect thrombocytes and leucocytes of birds. After their uptake by the vectors, the parasites’ sporozoites can persist within them for a long time and were seen up to 42 days post-infection in *Aedes aegypti* [6] (Figure 2). Attempts to experimentally infect canaries with *Lankesterella* strains isolated from other bird species did not succeed in the past [1,6], probably because of the high host specificity of *Lankesterella* parasites [6]. 

This study on *Isospora* and *Lankesterella* parasites aimed to (1) determine the infection rates of these parasites in wild passerine birds through molecular analyses, (2) investigate the phylogenetic relationships of these parasite lineages, (3) develop molecular probes for the specific detection of *Isospora* and *Lankesterella* parasites in formalin-fixed and paraffin-wax-embedded (FFPE) tissue samples using chromogenic in situ hybridization (CISH), and (4) analyze pathomorphological changes in FFPE tissue sections of infected birds to investigate the potential pathogenicity of these parasites.

## 2. Materials and Methods

### 2.1. Sample Collection

The present study comprised samples of 815 wild passeriform birds from Slovakia (302 individuals), Hungary (218), Austria (216), Lithuania (42), Switzerland (23), Bosnia and Herzegovina (7), and Russia (7). It included birds of 64 species from 24 families, most of which belonged to the Paridae (161 individuals), Sylviidae (145), Muscicapidae (135), Passeridae (57), Acrocephalidae (63), Hirundinidae (48), Phylloscopidae (42), Turdidae (40), and Fringillidae (38). The selection particularly included small passeriform birds, which were assumed to be more prone to infections with *Lankesterella* parasites due to their insectivorous feeding habits.

In addition to the samples from wild birds (*n* = 815), FFPE tissue samples from 15 deceased captive birds were included in this study. These birds were sent to the University of Veterinary Medicine Vienna (Vetmeduni) for pathological examination with the suspicion of being infected with ‘*Atoxoplasma*’ (=*Isospora*) parasites and comprised the following species: *Carduelis carduelis* (1), *Padda oryzivora* (1), *Passerina ciris* (1), *Serinus canaria* (3), *Spinus cucullatus* (3), *Spinus xanthogastrus* (3), and *Spinus yarrellii* (3). 

The material investigated originated from a range of different sources. The 302 Slovakian birds were captured using mist nets from 2017 to 2019 and in 2022 at the Drienovec Bird Ringing Station in the south-east part of the country near the Drienovec village as described previously [41]. Blood samples were taken by puncturing the brachial vein and stored in 70% ethanol at 4 °C until DNA extraction. Similarly, blood samples were taken from 128 birds captured at the Biological Station Illmitz in eastern Austria and used to prepare both blood spots on high-grade filter papers Whatman™ 903 for DNA extraction and blood smears on microscope slides. The same procedure was followed when taking blood samples from 15 bird patients housed at the Clinical Unit of Internal Medicine Small Animals of the Vetmeduni. Blood samples were also collected from seven birds caught with mist nets at the ‘Rybachy’ Biological Station on the Curonian spit in Kaliningrad oblast in 2014 and used to prepare blood spots and smears. All Hungarian (218) and Swiss (23) samples, and 41 of the Austrian ones, originated from dead birds collected for an Usutu virus monitoring study conducted by the Institutes of Pathology and Virology (Vetmeduni) from 2003 to 2007. These birds were dissected, and tissue samples were collected from the heart, lung, liver, spleen, kidney, brain, skeletal muscle, proventriculus, gizzard, and intestine, fixed in formalin, and embedded in paraffin wax. Brain tissue was stored at −80 °C for total RNA/DNA extraction [42]. FFPE tissue samples were also prepared from 32 dead birds collected during a Citizen Science project in eastern Austria in the summer of 2020. Liver and lung samples of the latter birds were frozen at −80° until DNA extraction [43]. Samples from Bosnia and Herzegovina were collected from seven dead birds submitted for routine pathological examinations at the Department of Pathology at the Sarajevo Faculty of Veterinary Medicine (University of Sarajevo) between 2017 and 2018, and DNA was extracted from frozen liver tissue. The 42 Lithuanian samples originated from birds caught with mist nets at the Ventės Ragas Ornithological station in Ventė Cape (western Lithuania) between 2018 and 2020. Blood spots and smears were prepared from fresh blood, and smears were fixed in absolute methanol and stained with 10% Giemsa solution [44]. FFPE tissue samples were prepared from various organs following euthanization by decapitation and dissection. In total, FFPE tissues from 367 birds were used for CISH-based parasite screenings. Most of the FFPE tissue blocks contained brain, heart, lung, liver, crop, proventriculus and gizzard, intestine, pancreas, kidney, ovary, testicle, skeletal muscle, bone marrow, spleen, and bursa of Fabricius tissues. However, some bird cadavers were not well preserved, and the FFPE blocks did not include the complete set of the above-listed organs.

### 2.2. DNA Extraction

DNA extraction of the Slovakian samples was performed at the Institute of Zoology of the Slovak Academy of Sciences in Bratislava. DNA was extracted using the QIAamp DNA Blood Mini Kit (QIAGEN GmbH, Hilden, Germany) following the manufacturer’s recommendation, resolved to a final concentration of ca. 100 ng/µL, and stored at −20 °C [41]. The DNA extractions of all other samples were performed at the Institute of Pathology, Vetmeduni Vienna. The total RNA/DNA of the samples collected for the Usutu virus project was extracted from 140 µL homogenates of brain tissue using the QIAamp Viral RNA Mini Kit (QIAGEN) [45]. In the case of the samples from the 15 deceased captive birds sent for pathological investigation to the Institute of Pathology, DNA was extracted from FFPE tissue blocks. In short, every 10 sections of 10 µm thickness were cut from the FFPE blocks, deparaffinized with limonene and ethanol, and then extracted with the QIAamp DNA Micro-Kit (QIAGEN) according to the manufacturer’s protocol. The DNA of the other samples was either extracted from blood spots on filter paper (ca. 1 cm^2^) or frozen liver and lung tissue (ca. 4 mm^3^) using the DNeasy Blood & Tissue Kit (QIAGEN GmbH) following the standard protocol. All DNA samples were stored at −20 °C either at the Institute of Zoology, Slovak Academy of Sciences, Bratislava, or the Institute of Pathology, Vetmeduni Vienna. 

### 2.3. Primer Design

A nested PCR assay was designed to sequence an 827 base pair (bp) section of the mitochondrial (mt) *cytochrome b* gene (*CytB*). The primers were designed based on an alignment comprising all published mitochondrial genomes of Eimeriidae. The primers were placed in conserved sequence regions and should allow amplification of this *CytB* fragment in most members of the family Eimeriidae. The first primer set, LankCytB_F1 (5′-GATCTCATTTACAATCATATCCATGTC-3′) and LankCytB_R1 (5′-TAGATTACGGTAAAGAATCTACCA-3′), amplifies a 1033 bp fragment (982 bp excl. primers). The second (nested) primer set, LankCytB_F2 (5′-GTGAAATTTCACATGCCTTTGC-3′) and LankCytB_R2 (5′-CTTCCTGAGGTAGTTGAGCTC-3′), amplifies an 870 bp fragment (827 bp excl. primers).

Since the DNA in FFPE samples is usually degraded to fragments of less than 400 bp, a second PCR assay was designed for amplifying three overlapping *CytB* fragments, which can be combined to an 823 bp *CytB* fragment covering roughly the same sequence region as the nested PCR assay. The primers I_CytB_F1 (5′-TGAAATGTCACATGCCTTTGC-3′) and I_CytB_R1 (5′-AGGACTATCTACATAAAAACCTCC-3′) [alternative for *Lankesterella*: L_CytB_R1 (5′-GTAGGATGATCTACATAGAATCCTC-3′)], I_CytB_F2 (5′-GGGTCAAATGAGCTTCTGG-3′) and I_CytB_R2 (5′-TATCTGGATGTGATAGTTCCAT-3′), and I_CytB_F3 (5′-TCCTATTAGCTCAATCATTCTTTG-3′) and I_CytB_R3 (5′-TGAGGTAATTGAGCTCCAAC-3′) amplify sections of 340 bp (295 bp excl. primers), 344 bp (303 bp), and 334 bp (290 bp), respectively. While the outer primers (I_CytB_F1 and I_CytB_R3) should also target the *CytB* of most Eimeriidae, the inner primers were designed based on the *Isospora CytB* sequences obtained in the present study only.

Another nested PCR assay was designed to sequence a 906 bp section of the mt *cytochrome c oxidase I* gene (*COI*) from a selection of approximately 70 samples containing 17 *Isospora* and 20 *Lankesterella* lineages obtained with the nested *CytB* PCR assay. The *COI* primers were also placed in sequence regions conserved within most members of the family Eimeriidae. The first primer set, LankCOI_F1 (5′-CTGCTGCAAACCATAAAGAATTAGG-3′) and LankCOI_R1 (5′-CAGGAATTCTACGTGGCATAACAT-3′), amplifies a 1335 bp fragment (1286 bp excl. primers). The second primer set, LankCOI_F2 (5′-TGGTTCAGGTATTGGTTGGA-3′) and LankCOI_R2 (5′-GACCATACTCTTAAGAATGGAGAATC-3′), amplifies a 906 bp fragment (860 bp excl. primers). 

The nested PCR assay by Chagas et al. [6] was used to sequence approx. 1000 bp fragments of the *18S* ribosomal RNA gene (*18S*) in a subset of samples containing a variety of *Isospora* and *Lankesterella* lineages. The first primer set, Cocc18S_n1F (5′-CAGCTTTCGACGGTATGGTATTGG-3′) and Cocc18S_n1R (5′-CAGACCTGTTATTGCCTCAAACTTCCT-3′), amplifies a fragment of 1135 bp. The nested primer set, Cocc18S_n2F (5′-GTATTGGCTTACCGTGGCAGTGAC-3′) and Cocc18S_n2R (5′-GCCTCAAACTTCCTTGCGTTAGACA-3′), amplifies a fragment of 1105 bp. The *18S* sequences were primarily used as a basis for designing the in situ hybridization probes to specifically target and differentiate parasites of the two genera.

### 2.4. PCR and Sequencing

All 830 individuals were screened for the presence of *Isospora* and *Lankesterella* parasites with the nested PCR approach targeting *CytB.* Birds for which only FFPE samples were available (*n* = 15) were additionally screened with the PCR assay using the three sets of overlapping primers. The *COI* and *18S* PCRs were performed on 48 samples. 

All PCRs were performed with the KAPA2G Fast HotStart PCR-Kit (Sigma-Aldrich, St. Louis, MO, USA) in 25 µL volumes containing 12.5 µL KAPA2G Fast HotStart polymerase mix, 8.5 µL AD, 1 µL MgCl_2_ (1.5 mM), each 1 µL primer (10 mM), and 1 µL template. The nested PCRs started with an initial denaturation for 2 min at 94 °C, followed by 20 (first PCR) or 35 (nested PCR) cycles with 15 s at 94 °C, 15 s at defined annealing temperatures for each primer set (see below), 30 s at 72 °C, and a final extension for 10 min at 72 °C. Each 1 µL PCR product of the first PCRs was used as a template for the nested PCRs. The annealing temperature for the two nested PCR assays (*CytB* and *COI*) was 53 °C, while the one for the PCRs targeting the short, overlapping *CytB* fragments was 48 °C. The PCRs targeting the 18S rDNA were performed at 56 °C annealing temperature.

The PCR products (3 µL) were visualized on 1% agarose gels stained with ROTI^®^GelStain (Carl Roth GmbH & Co, Karlsruhe, Germany) and imaged with a BioSens SC-Series 710 gel documentation system (GenXpress, Wiener Neudorf, Austria). Positive PCR products were sent to Microsynth Austria (Vienna, Austria) for purification and sequencing in both directions using the PCR primers. Raw forward and reverse sequences were aligned using MAFFT v.7 [46] applying the default settings, and the electropherograms were visually inspected with Bioedit v.7.0.8.0 [47]. Mixed infections were present in some samples, and positions with double peaks in the electropherograms were first coded with the corresponding ambiguity codes. Then, all *CytB* sequences were aligned and sorted by sequence similarity. Unphasing of sequences was straightforward because the individuals mostly contained one prevalent lineage present in other birds of the same species, or the lineages differed only by one or a few bp while one lineage was dominating. 

New haplotypes identified in the present study were assigned lineage names consisting of “i” for *Isospora* or “k” for *Lankesterella*, followed by the first three letters of the bird genera and bird species, and ascending numbers starting with “01”. The naming scheme was chosen following the system established for avian haemosporidian parasites in the MalAvi database (http://130.235.244.92/Malavi/; accessed on 1 December 2023 [48]), which summarizes DNA barcode sequences (476 bp *CytB* fragments) of parasite lineages and provides information on bird hosts, vectors, geographic regions, and literature. 

All sequences were uploaded to NCBI GenBank and can be retrieved under accession numbers PP666884–PP667037 (*CytB*), PP667038–PP667082 for (*COI*), and PP660150–PP660193 for (*18S*).

### 2.5. Phylogenetic Analysis

Phylogenetic trees were calculated with the *CytB* sequences of *Isospora* spp. and *Lankesterella* spp. and the *COI* sequences of *Isospora* spp. The only mt DNA sequences published for *Lankesterella* are the genomes of two *Lankesterella minima* lineages isolated from *Lithobates clamitans* frogs; therefore, no phylogenetic tree was calculated with the *Lankesterella COI* sequences. 

The alignments for the *CytB* trees of *Isospora* and *Lankesterella* contained 74 and 93 sequences, respectively (including those from mt genomes available on GenBank) and covered 827 bp. Sequences of *Lankesterella* sp. kSYLATR01 (AH0893) and *Isospora* sp. iPASDOM02 (AH1562) were used as outgroups. To visualize the distribution in host species, the sequences of these two alignments were not collapsed to haplotypes. 

*COI* sequences of *Isospora* parasites have been retrieved from various species of wild and captive birds. We included all *Isospora COI* sequences from GenBank, which covered the selected 648 bp *COI* fragment and contained less than two ambiguity characters. The initial alignment contained 217 sequences from GenBank and 24 obtained in the present study. The sequences were collapsed to 194 unique haplotypes with DAMBE v.7.0.5.1 [49], and a sequence of *Lankesterella* sp. kERIRUB01 (AH0896) was included as an outgroup.

Phylogenetic trees were also calculated with partitioned datasets containing the concatenated *CytB* (827 bp) and *COI* (860 bp) sequences of *Isospora* spp. and *Lankesterella* spp. The alignment for *Isospora* spp. contained the sequences of 18 different lineages and the outgroup *Lankesterella* sp. kSYLATR01 (SK162/19). The alignment of *Lankesterella* spp. contained sequences of 22 different lineages and the outgroup *Isospora* sp. iPASDOM02 (AH1553). The trees contain fewer haplotypes than the *CytB* trees because we were not able to sequence the *COI* sequences for all *CytB* lineages.

The best-fit substitution models according to the corrected Akaike Information Criterion were evaluated using IQ-TREE [50], resulting in the model GTR+G+I for all three alignments. Maximum likelihood (ML) majority-rule consensus trees were calculated with W-IQ-TREE by performing 10,000 replicates. Bayesian Inference (BI) trees were calculated with MrBayes v.3.2 [51], running 5 million generations (two runs each with four chains, one of which was heated) and sampling every thousandth tree. The first 25% of trees were discarded as burn-in, and a 50% majority rule consensus tree was calculated from the remaining 3750 trees each.

All alignments were visually inspected using Bioedit v.7.0.8.0 [47]. The phylogenetic trees were graphically prepared using FigTree v.1.4.4 (http://tree.bio.ed.ac.uk/software/figtree/; accessed on 14 March 2023; Andrew Rambaut) and finalized with Adobe Illustrator CC v.2015 (Adobe Inc., San José, CA, USA).

### 2.6. Genetic Distances between Isospora and Lankesterella CytB Lineages

Uncorrected genetic distances between the *Isospora* and *Lankesterella CytB* (823 bp) lineages detected in the present study were calculated using MEGA v.10.2.2 [52]. The distances were calculated separately based on the alignments containing 31 *Isospora* and 27 *Lankesterella* lineages. Sequences of *Lankesterella* sp. kPASDOM02 and *Isospora* sp. iSYLATR01 were included for comparison. The alignments were trimmed to 823 bp, the length of the 4 bp shorter *Isospora* sequences obtained from the FFPE samples.

### 2.7. Chromogenic In Situ Hybridization

To detect *Isospora* and *Lankesterella* parasite stages by CISH, two probes were designed based on an alignment containing all available *18S* sequences of *Isospora* and *Lankesterella* (including 28 sequences generated in the present study), most other Eimeriidae, and several bird species. The probes were commercially synthesized by Microsynth Austria (Vienna, Austria) including digoxigenin labels at the 3′ ends. The probes are complementary to the DNA sequence of the *18S* rRNA gene and directly target the *18S* rRNA. The probe Iso18S_ISH (5′-GGAAGAAAGCCGAGGGCAAACCAGGTGC-3′) binds the *18S* rRNA of all avian *Isospora* lineages with a maximum of one or two mismatches. Moreover, it binds the *18S* rRNA of numerous closely related *Eimeria* species isolated from Muridae, Sciuridae, Bovidae, Leporidae, and galliform birds. It was not possible to design a probe specific to avian *Isospora* parasites because their *18S* sequences differed only in a few bp from those of many other Eimeriidae. The probe Lan18S_ISH (5′-GGAAATATGCCACAATCCATCCTC-3′) binds the *18S* rRNA of all published avian *Lankesterella* lineages with a maximum of one or two mismatches. The probe is specific to this parasite group and does not match the *18S* rRNA of other Eimeriidae, including *Lankesterella* parasites found in American lizard species by Megía-Palma et al. [37].

The specificity of the probes was checked in silico. AmplifX v.2.0.7 (Nicolas Jullien, Aix-Marseille Univ., CNRS, INP, Marseille, France; https://inp.univ-amu.fr/en/amplifx-manage-test-and-design-your-primers-for-pcr; accessed on 3 September 2021) was used to check the quality of the newly designed probes. All probes were blasted against genomes of apicomplexan parasites and birds in NCBI GenBank to exclude unintentional binding. To confirm the specificity of the probes, cross-checking was also performed by CISH to rule out the chance that the probe designed for *Isospora* binds to *Lankesterella* in the tissue and vice versa, potentially causing false-positive results (Supplementary File Figure S1). 

CISH was performed on all Eimeriidae PCR-positive birds of which FFPE samples were available (*n* = 53). The procedure followed a previously established CISH protocol [53] with some modifications. Paraffin-wax-embedded tissue samples were sectioned (2–3 µm) and placed on Superfrost Plus slides (Menzel-Gläser, Braunschweig, Germany). After drying, sections were dewaxed in orange terpene (SAV Liquid Production GmbH, Flintsbach am Inn, Germany) and rehydrated in a series of graded alcohols (100%, 96% and 70%) and distilled water. Proteolytic treatment was performed with Proteinase K 3.3 µg/mL (Roche Diagnostics, Vienna, Austria) in Tris-buffered saline (pH 7.4) at 37 °C for 40 min. After proteolysis, the slides were rinsed with distilled water and dehydrated in 96% and 100% ethanol, followed by air-drying. The hybridization mixture consisted of distilled water, 20× standard saline citrate buffer (SSC), formamide, 50× Denhardt’s solution, herring sperm DNA, dextran sulfate, and the appropriate oligonucleotide probe (10 ng/100 µL). Slides were covered with the hybridization mixture and incubated at 95 °C for 6 min and then immediately cooled down on crushed ice and hybridized overnight in a humid chamber at 40 °C. On the second day, three stringency washing procedures (2 × SSC, 1 × SSC, and 0.1 × SSC, 10 min each at room temperature) were carried out to remove unspecific bindings. For immunological detection of hybrids, slides were covered with an equilibration buffer containing distilled water, buffer I (2×) (1M Tris-HCl 20%, 5M NaCl 6%, and DEPC-treated water), normal goat serum (VECS-1000, Szabo-Scandic, Vienna, Austria), and Triton X-100 10% (Merck, Darmstadt, Germany) for 30 min at room temperature. Afterward, slides were incubated with anti-digoxigenin-AP Fab fragments (#11093274910, Merck KgA, Darmstadt, Germany) at a concentration of 1:200 for 1 h at room temperature followed by two 15 min washing steps in buffer I (1×) and briefly in buffer III (100 mM Tris-HCl, 100 mM NaCl, 50 mM MgCl_2_ in DEPC-treated water, final pH 9.5). To visualize the probe–parasite hybrids, the chromogenic substrates 4.5 µL/mL NBT (4-nitro blue tetrazolium) (Roche Diagnostics) and 3.5 µL/mL BCIP (5-bromo-4-chloro-3-indolyl phosphate) (Roche Diagnostics) were mixed with 2.5 µL/mL Levamisol (Merck) in buffer III and applied to the slides. After incubation in a dark humid chamber for at least 60 min at room temperature, the chromogenic reaction was stopped by placing the slides in TE buffer (pH 8.0) for 10 min. As a final step, the slides were counterstained with hematoxylin, Gill 3 (Merck, Darmstadt, Germany), and mounted with Aquatex (Merck, Darmstadt, Germany).

### 2.8. Microscopic Examination

Hematoxylin–eosin (HE)-stained and CISH-treated tissue sections were evaluated by bright field microscopy using 100×, 200×, 400×, and 1000× magnifications. Microphotographs were taken using an Olympus UC90 camera attached to an Olympus BX51 microscope (Olympus Europa, Hamburg, Germany).

All tissue samples were examined to determine the degree of parasite burden following the semiquantitative scoring system developed as part of this study: negative (−; no signal in sections detected), low-grade (+), moderate (++), and high-grade (+++). 

The received score was determined by using a reward point system in three steps. First, points were assigned regarding the signal’s distribution. Tissues with an oligofocal distribution received 1 point. Tissues that exhibited multifocal signals received 2 points, and 3 points corresponded to a diffuse distribution. In the next step, the number of signals per foci was recorded. Single signals or a cluster of up to 20 signals resulted in 1 point. Two points were given if a cluster showed between 20 and 40 signals, and 3 points if more than 40 but less than 60 signals were seen. If there were more than 60 signals per cluster, 4 points were given. Finally, the points awarded were added, resulting in a final score, distinguishing between + (1–3 points; low-grade parasite burden), ++ (4–5 points; moderate parasite burden), and +++ (≥6 points; high-grade parasite burden).

Corresponding HE-stained tissue sections were examined to confirm the presence of parasites based on references [10,18,54]. In addition, pathological changes in all tissue samples were recorded to evaluate potential pathogenicity. It should be noted that many organs showed autolytic changes of varying severity, which led to limited histological evaluation in some cases. 

### 2.9. Graphical Representation of the Parasites’ Life Cycles

The illustration software BioRender (https://www.biorender.com) was used for the graphical creation of the presumed parasites’ life cycles based on descriptions in [6,18].

## 3. Results

### 3.1. Infection Rates

Based on PCRs and *CytB* sequencing results, the overall infection rate for *Isospora* in the wild birds was 5.8% (47/815). Double and triple infections with different *Isospora* lineages were detected in seven and two individuals, respectively. In total, 26 *Isospora CytB* lineages were recorded, most of which were specific to one or two host species. The *Isospora* infection rates differed strongly between the most frequently sampled bird species, with 50% in *Passer domesticus* (*n* = 46), 5.8% in *Erithacus rubecula* (*n* = 104), 3.5% in *Parus major* (*n* = 86), 2.5% in *Sylvia atricapilla* (*n* = 118), and no records in *Cyanistes caeruleus* (*n* = 65) and *Hirundo rustica* (*n* = 43). High infection rates were also recorded in *Luscinia luscinia* (100%), *Phoenicurus ochruros* (62.5%), and *Regulus regulus* (27.3%); however, bird sample sizes of these species were small, with one to eleven individuals (Table 1). 

Moreover, 5 of the 15 captive birds of which only FFPE samples were available were confirmed positive for *Isospora* spp.: *P. ciris* (1/1), *P. oryzivora* (1/1), *C. carduelis* (1), *S. canaria* (1/3), and *S. yarrellii* (1/3). They featured five lineages, four of which were not found in the wild bird samples.

The overall infection rate for *Lankesterella* was 10.7% (87/815), and only two individuals featured double infections. In total, 25 *Lankesterella CytB* lineages were recorded, which were mostly restricted to single host species as well. The infection rates also differed strongly with 21.2% in *E. rubecula*, 17.8% in *S. atricapilla*, 14.0% in *H. rustica*, 7.0% in *P. major*, 6.5% in *P. domesticus*, and 1.5% in *C. caeruleus*. The highest infection rates were recorded in *Motacilla alba* (50%), *Ficedula hypoleuca* (45.5%), *Lanius collurio* (42.9%), and *Acrocephalus schoenobaenus* (37.5%) (Table 1).

In addition to *CytB* sequences, *COI* sequences were obtained from 20 of the 26 *Isospora* lineages (from wild birds) and from 23 of the 25 *Lankesterella* lineages. The *18S* sequences were obtained from 18 *Isospora* and 10 *Lankesterella* lineages. In both genera, *CytB* sequences of some lineages differed by one to a few bp, but the corresponding *COI* sequences were identical. This was the case for the *Isospora* lineage pairs iREGREG01 and iREGREG02, iPASDOM03 and iPASDOM06, and iEMBCIR01 and iEMBCIR02, which differed by 5 bp, 2 bp, and 1 bp, respectively. Similarly, the *Lankesterella* lineages kPASDOM02 and kPASDOM03 and kPHYCOL01 and kPHYCOL02 both differed by 1 bp in *CytB*, but the *COI* sequences were identical.

### 3.2. Phylogeny

**Figure 3:**
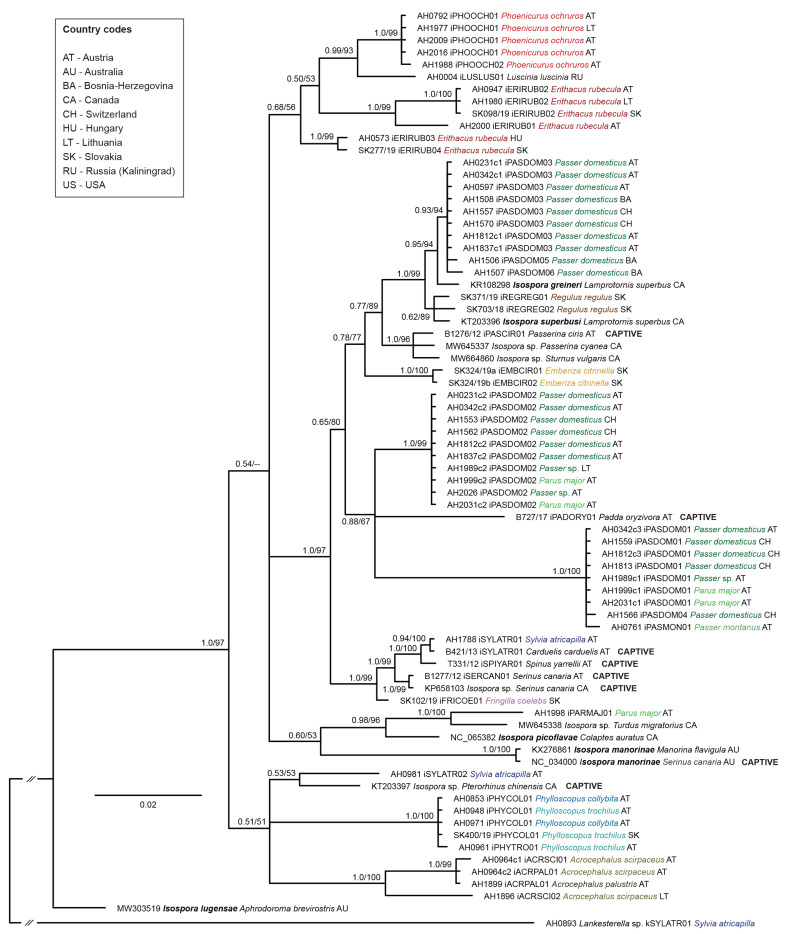
Bayesian Inference tree of partial *cytochrome b* sequences (827 bp) of avian *Isospora* parasites. Posterior probabilities and maximum likelihood bootstrap values are indicated at all nodes. The scale bar indicates the expected mean number of substitutions per site according to the model of sequence evolution applied. A sequence of *Lankesterella* sp. kSYLATR01 from *Sylvia atricapilla* was included as an outgroup. Different colors are provided for the host species investigated in the present study to enhance comparability.

The *Isospora CytB* tree contained 43 unique lineages (including sequences from ten mt genomes published on GenBank) (Figure 3). The highest *p*-distance measured between the *Isospora* lineages was 8.0%, and the mean distance was 5.0%. A lineage of *Isospora lugensae* (GenBank accession MW303519) formed the sister group to a major clade containing all other *Isospora* lineages, which clustered into several subclades. The subclades mainly contained lineages retrieved from bird species of the same family, suggesting the *Isospora* lineages had high host specificity. The birds kept in captivity (FFPE samples) featured five *Isospora* lineages, two of which were found in other birds. The lineage iSYLATR01 from a captive *C. carduelis* (B421/13) was also detected in *S. atricapilla* (AH1788) from Austria and differed by 0.4% from lineage iSPIYAR01 from *S. yarrellii* (T331/12), and one *S. canaria* (B1277/12) was infected with the lineage iSERCAN01, which previously was isolated from *S. canaria* (KP658103) in Canada [55]. All the latter lineages clustered into a highly supported clade together with lineage iFRICOE01 from *Fringilla coelebs* in Slovakia, which also belongs to the Fringillidae. The lineage iPASCIR01 from *P. ciris* (B1276/12) differed by 6 bp and 7 bp from *Isospora* lineages found in *Passerina cyanea* (MW645337) and *Sturnus vulgaris* (MW664860) from Canada, respectively (published on GenBank only). The subclade containing the three latter lineages fell in a clade with other lineages mostly isolated from Passeridae and Sturnidae. The lineage iPADORY01 from a captive *P. oryzivora* formed an independent branch and clustered with two subclades containing parasite lineages from Passeridae and Paridae. Genetic p-distances between the *Isospora CytB* lineages (823 bp) detected in the present study are provided in Supplementary File Table S1.

**Figure 4:**
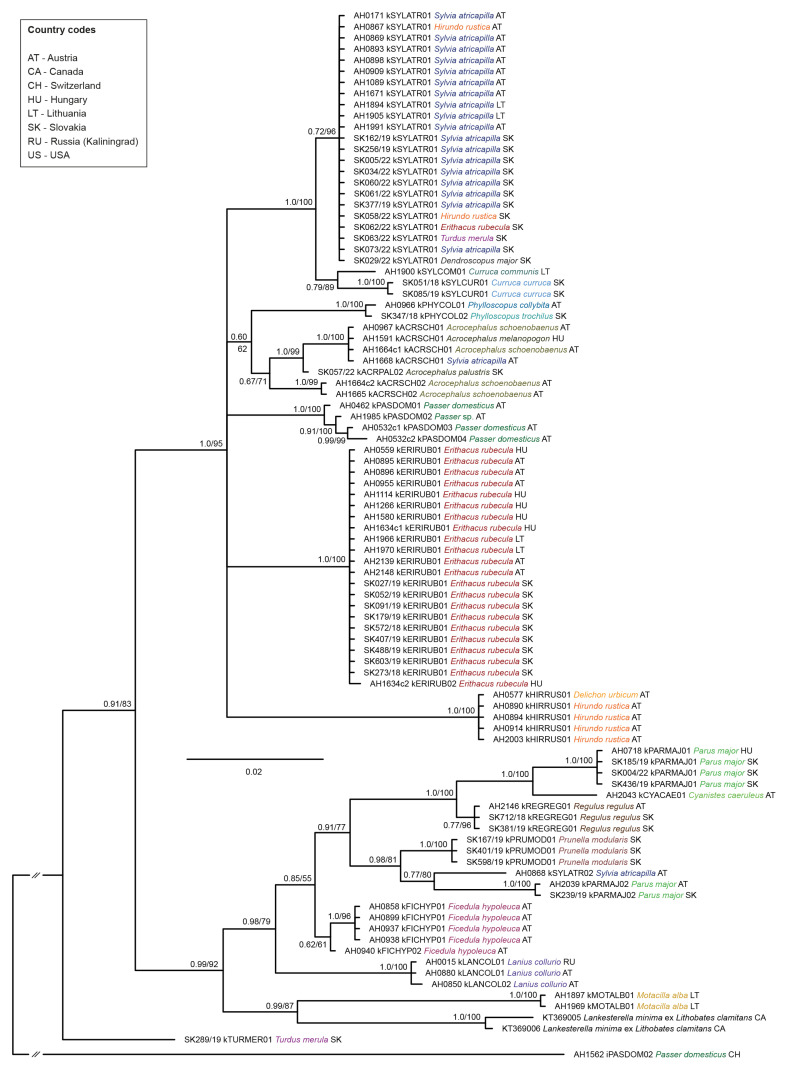
Bayesian Inference tree of partial *cytochrome b* sequences (827 bp) of avian and amphibian *Lankesterella* parasites. Posterior probabilities and maximum likelihood bootstrap values are indicated at all nodes. The scale bar indicates the expected mean number of substitutions per site according to the model of sequence evolution applied. A sequence of *Isospora* sp. iPASDOM02 from *Passer domesticus* was included as an outgroup. Different colors are provided for the host species investigated in the present study to enhance comparability.

The *Lankesterella CytB* tree contained 29 unique lineages (including sequences of two mt genomes of *L. minima* isolated from *L. clamitans* (Figure 4). The highest *p*-distance between different *Lankesterella* lineages was 8.6%, and the mean distance was 5.0%. Most lineages were restricted to single bird species; however, lineage kSYLATR01 was detected in four bird species: *S. atricapilla*, *H. rustica*, *E. rubecula*, and *Turdus merula*. The lineage kTURMER01 from *T. merula* in Slovakia was the sister group to all other *Lankesterella* lineages, which were grouped into two well-supported clades, each containing several subclades. Interestingly, the two *L. minima* lineages from the green frog *L. clamitans* clustered in a clade with lineage kMOTALB01 from *M. alba* in Lithuania. Genetic p-distances between the *Lankesterella CytB* lineages (823 bp) detected in the present study are provided in Supplementary File Table S2.

A phylogenetic tree was also calculated with the *COI* sequences of *Isospora* spp. from the present study and sequences published from other wild bird species on GenBank (Supplementary File Figure S2). None of the *Isospora COI* lineages was detected in previous studies. The tree contained a total of 193 different *Isospora* lineages, most of which (>140) were published by Kubiski et al. [24], who screened samples from captive and wild birds collected by the San Diego Zoo Wildlife Alliance. 

The phylogenetic trees calculated with the concatenated *COI* and *CytB* sequences contained 18 *Isospora* and 22 *Lankesterella* lineages. The topologies resembled those in the *CytB* trees, but the support values were also low at the deeper nodes (Supplementary File Figure S3). Future phylogenetic studies would benefit from analyzing the complete mt genomes and the more variable nuclear coding genes.

### 3.3. Chromogenic In Situ Hybridization

Among the birds that were PCR-positive for *Isospora* (*n* = 53), FFPE tissue samples were available for subsequent CISH in 26 cases. In the *Lankesterella*-infected birds (*n* = 87), FFPE tissue samples were available for CISH from 20 individuals. However, FFPE tissue samples did not always contain the same set of organs for every individual. In both parasite genera, the brain was most frequently part of the organ set and was only missing in one case each. Tissue samples from the heart, liver or kidney were present for at least 80% of these individuals. Lung, stomach (proventriculus and/or ventriculus), skeletal muscle, and spleen samples were available for CISH in at least half of the *Isospora*- and *Lankesterella*-infected birds. In 54% of *Isospora*-infected birds, the intestine was also part of the tissue samples. In many birds, the esophagus, pancreas, gonads, trachea, and bursa of Fabricius were not available. Only in one bird infected with *Isospora*, tissue samples also included crop and bone marrow. In 34 of 46 samples (74%), *Isospora* or *Lankesterella* were successfully identified by the dark-purple-to-black color reaction using the respective genus-specific probes. In both *Isospora*- and *Lankesterella*-infected birds, signals appeared in various organs, including the heart, lung, liver, spleen, kidney, brain, skeletal muscle, proventriculus and gizzard, intestine, esophagus, testicle, ovary, and pancreas. The distribution of detected signals varied between the individual hosts, ranging from single infected organs to a generalized infection and also between the two parasite genera, which is described in more detail below. 

### 3.4. Detection of Isospora by CISH

In 22 out of 26 (85%) examined *Isospora*-infected birds, CISH signals were found, with the distribution and abundance of signals in the different organs varying between individuals (Table 2). In most cases, systemic infections were observed, which appeared mild in most (*P. major*, *P. ochruros*) and severe in some cases (*P. ciris*, *P. oryzivora*, *S. yarrellii*, *P. domesticus*). A low-grade parasite burden in at least one organ was found in twelve birds (46%). In only two cases (8%), moderate numbers of stained parasites in at least one organ were detected. Eight birds (31%) showed a high-grade parasite burden.

*Isospora* parasites frequently appeared in the spleen (87%), intestine (79%), and liver (71%) of CISH-examined birds (Table 2), followed by the kidney (45%), gizzard and/or proventriculus (41%), and lung (40%). Signals were less commonly observed in the skeletal muscle (33%) and heart (29%). In three cases, signals were present in the brain (12%). Parasites were also found in the bone marrow, crop, pancreas, ovary, and esophagus. However, the sample size of individuals from which these organs were available for CISH was small (Table 2), ranging from one to seven. No signals were detected in the testicle, trachea, and bursa of Fabricius.

**Figure 5:**
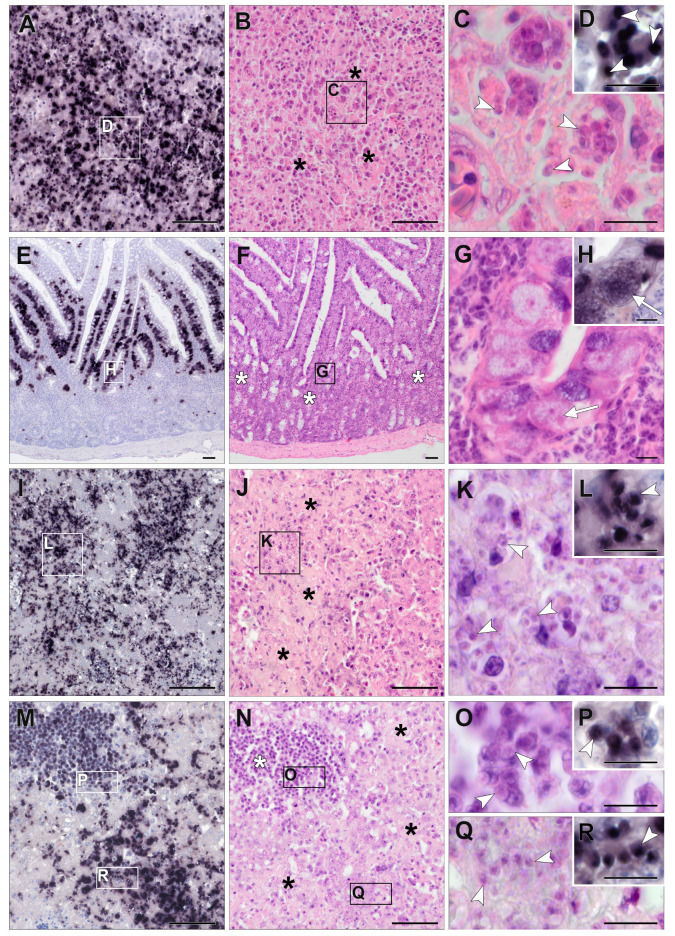
Histological sections of the spleen (**A**–**D**) of a captive *Passerina ciris* (B1276/12), intestine (**E**–**H**) of a wild *E. rubecula* (AH1980), and liver (**I**–**R**) of a captive *Padda oryzivora* (B727/17). The images in the right-hand column show higher magnifications of the regions marked by rectangles in the left columns. Note that the CISH images in the right column show a smaller field of view than contained in the rectangles. CISH using an *Isospora*-specific probe showed distinct purple signals, confirming the parasites’ presence (**A**,**D**,**E**,**H**,**I**,**L**,**M**,**P**,**R**). Multiple *Isospora* parasites (white arrowheads, presumably merozoites) were seen in the spleen (**C**) and liver (**K**,**O**,**Q**) in corresponding HE-stained sections. Note the *Isospora* parasites (white arrowheads) within the cytoplasm of mononuclear cells indenting the nuclei (**K**,**O**). Multifocal necrosis (black asterisks) in the spleen (**B**) and liver (**J**,**N**) was associated with *Isospora* parasites. With CISH, *Isospora* parasites were easily detected by their purple staining within enterocytes (**H**, white arrow). The corresponding HE-stained section showed *Isospora* parasites of different shapes and sizes (**G**, white arrow, presumably macrogamont). Inflammatory response (**F**, white asterisks) was observed in the lamina propria mucosae of the intestine. Scale bars: (**A**,**B**,**E**,**F**,**I**,**J**,**M**,**N**) 50 µm; (**C**,**D**,**G**,**H**,**K**,**L**,**O**,**R**) 10 µm.

High parasite burdens, determined using the CISH score, were mostly found in the liver and spleen (Figure 5) of captive *P. ciris*, *P. oryzivora*, *C. carduelis*, *S. yarrellii,* and three wild *P. domesticus*. Detected signals were mostly roundish to oval, varied in size, and were assumed to be located in mononuclear cells, corresponding to extraintestinal merogony stages (=merozoites) (Figure 5, Supplementary File Figure S4). The bone marrow of the aforementioned *P. ciris* (B1276/12)—the only individual including bone marrow in the organ set—also revealed a high parasite burden. Furthermore, a high parasite burden was detected in the intestines of each *P. oryzivora* and *E. rubecula*, with much larger, roundish to oval or polygonal signals of varying intensity located in epithelial cells, corresponding to micro- and macrogamonts (Figure 5). A moderate parasite burden of presumably merozoites was found in the lung, kidney, gizzard and proventriculus (Supplementary File Figure S4), ovary, and crop of various bird species. The heart, brain, skeletal muscles, esophagus, and pancreas of infected birds were the least affected and showed low parasite burden (Supplementary File Figure S4). This was particularly evident in Passeridae.

Relatively small, roundish to oval signals appeared in the lumina of larger vessels or capillaries of various organs and were therefore evaluated as parasitic blood stages. Signals were more frequently found in endothelial cells or closely associated with the endothelia of differently sized blood vessels; however, their cellular location (intra- or extracellular, intra- or extraluminal) could not be determined with certainty.

In the case of *Isospora*-infected birds, signals could often be distinguished either as tissue stages or blood stages. However, for some signals, it could not be determined with absolute confidence whether they were representing tissue or blood stages due to the low intensity of the signal or poor preservation status of the tissue. 

### 3.5. Detection of Lankesterella by CISH

In 12 of 20 (60%) *Lankesterella*-infected birds, CISH signals were detected (Table 3). In those birds, a low-grade parasite burden in at least one organ was observed.

**Figure 6:**
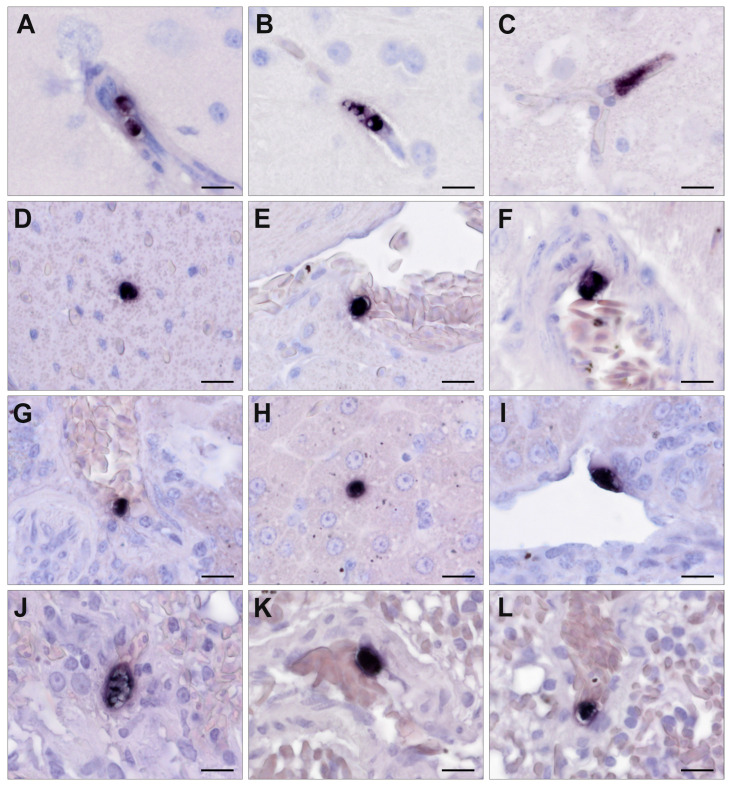
Parasite stages of *Lankesterella* spp. in infected wild birds, identified by CISH in brain (**A**–**C**), heart (**D**–**F**), liver (**G**–**I**), and lung (**J**–**L**) sections. The tissue sections originate from a *Motacilla alba* (AH1897) with the exception of (**C**,**F**). (**C**) belongs to the brain of a *Hirundo rustica* (AH2003) and (**F**) to the heart of another *M. alba* (AH1969). (**A**,**B**) Relatively small parasites within capillaries were discernible by their purple staining. (**C**,**E**–**G**,**I**,**K**,**L**) Roundish to oval signals appeared to be closely associated with endothelia of different-sized blood vessels. (**D**,**H**,**J**) Single signals of varying size and color intensity, in which the exact cellular localization of the parasite could not be precisely determined in the respective organ. Scale bars: 10 µm.

*Lankesterella* parasites frequently appeared in the spleen (44%), lung (36%), and brain (32%) of CISH-examined birds (Table 3). The heart, liver, and kidney were affected in 19% of examined birds. Signals were also observed in the gizzard, including the proventriculus (20%), and skeletal muscle (14%). In addition, signals were observed in testicles (40%) and intestines (14%), but again, the number of birds in which these two organs were part of the examined set of organs was rather low, being five and seven individuals, respectively (Table 3). Signals were absent in the trachea, esophagus, ovary, bursa of Fabricius, and pancreas. FFPE tissue samples tested for *Lankesterella* parasites did not contain bone marrow or crop tissue. The detected signals varied in terms of shape (roundish to elongated oval) and size (Figure 6). Similar to the CISH results for *Isospora*-infected birds, many signals appeared to be closely associated with the endothelia of differently sized blood vessels, which was observed in various organs of several birds. Some relatively small signals appeared to be located in the lumina of blood vessels indicating blood stages. However, the exact cellular location of the signals could often not be determined, partly due to low signal intensity or autolytic changes.

### 3.6. Histopathology of Isospora Infections

Histological evaluation of HE-stained tissue samples was often difficult due to autolytic changes. Therefore, reliable histologic identification of *Isospora* stages was not always possible. Histological lesions were mainly observed in the liver, spleen, intestine, proventriculus, and lung, with lymphocytic to lymphohistiocytic or histiocytic inflammation predominantly in a focal to multifocal distribution. In various organs of good preservation status, large numbers of *Isospora* parasites (presumably merozoites) were recognizable intralesionally in mononuclear cells like lymphocytes and macrophages (Figure 5, Supplementary File Figure S4). The number of parasites often appeared to correlate with the severity of lesions. In addition, mild to severe, oligo- to multifocal acute necrosis associated with recognizable parasites was observed in the liver and spleen (Figure 5). In some cases, intestinal epithelia contained large numbers of *Isospora* parasites (presumably micro- and macrogamonts) (Figure 5). In the intestine, lymphocytic inflammatory cells were seen mainly in the lamina propria (Figure 5). Low-grade to moderate proventriculitis, ventriculitis, or both were seen in several cases, sometimes accompanied by parasitic stages (presumably merozoites, Supplementary File Figure S4). Focal low-grade to moderate serositis of the proventriculus, gizzard, and kidney with concomitant parasites (presumably merozoites) was noted in single cases (Supplementary File Figure S4). The kidney and ovary showed low- to moderate-grade inflammation with associated parasites in individual cases. Inside the wall of a blood vessel, parasites (presumably merozoites) were detected in the brain of one bird (Supplementary File Figure S4). In another case, many parasites were identified in bone marrow. Low-grade, lymphocytic to granulomatous myocarditis was observed in a few birds, but concomitant parasites were absent. 

### 3.7. Histopathology of Lankesterella Infections

Several birds showed pathological lesions in various HE-stained tissues; however, these lesions were not accompanied by CISH signals for *Lankesterella*. Furthermore, parasitic stages of *Lankesterella* matching the CISH signals’ locations were not identified in subsequent HE-stained sections, probably due to their small size.

The intestines of three birds showed focal to multifocal lymphocytic enteritis, with moderate to severe inflammation in two cases and only mild enteritis in the third bird. In the intestinal epithelium of these three individuals, some roundish, protozoan-like objects that were not stained with the *Lankesterella*-specific CISH probe were found. Cross-testing of one of these birds using the *Isospora*-specific probe revealed positive staining of these structures, indicating co-infection (Supplementary File Figure S5). 

## 4. Discussion

This study presents the first systematic exploration of *Isospora* and *Lankesterella* parasites in wild songbirds, based on a sample including 815 individuals. The overall infection rates for *Isospora* and *Lankesterella* were 5.8% and 10.7%, respectively. The analysis of partial *CytB* sequences revealed 26 *Isospora* and 25 *Lankesterella* lineages. Most of the parasite lineages were detected exclusively in one host species, supporting high host specificity. In addition to the partial *CytB* sequences, partial *COI* and *18S* sequences were obtained from a subset of samples covering most parasite lineages. Specific probes were designed for the detection of *Isospora* and *Lankesterella* parasites in tissues using CISH. Our findings revealed that *Isospora* most commonly infects the spleen, intestine, and liver, while *Lankesterella* is predominantly located in the spleen, lung, and brain.

*Passer domesticus* was the host species showing the highest infection rate of *Isospora*, whereas *E. rubecula* and *S. atricapilla* had the highest infection rates of *Lankesterella* parasites. This difference in host preference is likely attributed to the behavioral aspects of the hosts and the life cycles of the parasites. Since *Isospora* is transmitted through feces [12], which are most commonly found on the ground, it is conceivable that key hosts for this parasite would be species primarily moving and feeding on the ground. Hence, we observed high infection rates in ground-dwelling species such as *P. domesticus* and lower infection rates in species like *E. rubecula* or *S. atricapilla*. A completely different transmission route is observed for *Lankesterella*, which is most likely not transmitted through feces but by eating the infected vector [6].

Reports of avian *Isospora* infections either originate from captive birds [19,20,22,56,57] or single individuals of deceased wild birds [26,27], and one study investigated both captive and wild birds [24]. Only a few studies exclusively analyzed infection rates of *Isospora* in larger groups of free-ranging wild birds [25,54,58,59]. Gill and Paperna [54] examined fecal samples of wild *P. domesticus* and found *Isospora* oocysts by floatation in the feces of birds trapped in the Jordan valley (90/124), the coastal plain south of Tel Aviv (27/32), and in the desert region in southern Israel (17/22), revealing infection rates of over 70% in each region. In our study, *P. domesticus* was also frequently infected with an infection rate of 50%, which could be related to its habits as a ground-dwelling species. In Western Australia, fecal samples from 34 *Corvus coronoides* were examined by flotation and sequence analysis, resulting in an *Isospora* infection rate of 44.1% [25]. Svobodová [59] analyzed fecal samples of 571 individuals belonging to 46 species of passeriform birds caught in different areas of the Czech Republic and detected a considerably higher overall infection rate for *Isospora*, 36.4%, by direct light microscopy of sporulated oocysts as compared to our study (6%). *Hippolais icterina* of the family Acrocephalidae had the highest infection rate in the study presented in [59], namely 79%. We also found Acrocephalidae species to be infected, namely *Acrocephalus scirpaceus* (9.1%) and *Acrocephalus palustris* (5.3%), while the only *H. icterina* individual examined was uninfected.

The infection rate of *Lankesterella* in the present study was only slightly higher, at 10.7%, than the one reported by Chagas et al. [6], namely 8.6%. In our study, birds belonging to the family Muscicapidae and Sylviidae presented a higher infection rate of *Lankesterella*; however, Chagas et al. [6] analyzed a small number of Sylviidae and no Muscicapidae. Saravana Bhavan Venkatachalam et al. [40] analyzed several bird species but did not report the rates of infection. *Acrocephalus schoenobaenus* was sampled in different studies at different localities. The *Lankesterella* infection rate was 47% in Poland [7], 17.2% in Lithuania [6], and 37.5% in the present study. Merino et al. [9] investigated *C. caeruleus* in Spain for *Lankesterella* and reported an infection rate of 31.2%, which is much higher than the one reported in our study (1.5%), and no infections were found in the three individuals investigated by Chagas et al. [6]. The closely related *P. major* presented a higher infection rate in our study (7%) than that found by Chagas et al. [6]. Such differences between the rates of infection are still not completely understood and should be better investigated. The sampling season might partially explain these discrepancies since juveniles might be less frequently infected than adults [6]. Additionally, the availability of vectors is also a determinant for the rate of infections, and the complete life cycle of *Lankesterella* is still unknown. 

The phylogenetic analyses demonstrated that avian *Isospora* parasites are a reciprocally monophyletic group based on their mt *COI* and *CytB* genes. This study revealed 43 distinct *Isospora* lineages, with a high degree of host specificity. The distribution of lineages seems to be rather correlated with host species than geographic regions. Our results are generally in line with those of Kubiski et al. [24], who emphasized the host specificity of *Isospora* lineages, highlighting co-evolutionary aspects of these interactions. In the present study, *Isospora* sequences generally were specific to one host species or closely related species of the same genus. However, for several lineages, the phylogenetic analysis revealed a broader host range including species from different families; e.g., the lineages iPASDOM02 and iPASDOM01 both infected *P. domesticus* and *P. major*. The presence of the *Isospora* lineage iSYLATR01 in *S. atricapilla* (family Sylviidae) and *C. carduelis* also illustrates the parasite’s ability to infect hosts across different bird families. This suggests a broader host range than previously understood, potentially indicating opportunistic host switching or more flexible parasite–host interactions. Insectivorous bird species were found to be more likely infected with *Lankesterella* parasites. However, most of these bird species also contained *Isospora* parasites (Figure 3 and Figure 4).

Another explanation for the detection of the same lineage in different bird hosts could be pseudoparasitism. Trefancová et al. [60] highlighted the phenomenon of pseudoparasitism in *Isospora*; they found oocysts of avian *Isospora* parasites in the feces of bank voles and yellow-necked mice. Additional data are needed to verify our observations. 

The phylogenetic analysis of *Lankesterella CytB* sequences revealed 29 distinct lineages differing by a maximum of 8.6%, highlighting a significant genetic diversity within the genus. This study found that most lineages were specific to one host, except for the lineage kSYLATR01, which was found in multiple bird species from different families (*S. atricapilla*, *H. rustica*, *E. rubecula*, and *T. merula*), indicating complex host–parasite dynamics. This pattern aligns with the findings of Chagas et al. [6] and Saravana Bhavan Venkatachalam et al. [40]. The phylogenetic analysis also revealed that some bird species featured *Lankesterella* lineages from different clades. Notably, the lineage kPARMAJ01 exhibits a sister lineage relationship with kCYACAE01 in *C. caeruleus*, a pattern not observed by Saravana Bhavan Venkatachalam et al. [40]. However, Saravana Bhavan Venkatachalam et al. [40] analyzed partial *18S* sequences, while our study mainly focused on *CytB*. The distinct positioning of kTURMER01 as a sister group to all other lineages indicates a potential early divergence within the genus. This branching pattern resonates with the evolutionary insights provided by Megía-Palma et al. [61] for the family Eimeriidae, which includes both *Isospora* and *Lankesterella*. Furthermore, the finding that the frog parasite *L. minima* and the lineage kMOTALB01 from *M. alba* clustered together highlights the potential for cross-species transmission and evolutionary adaptability within *Lankesterella*. This observation is in line with the broader understanding of parasite–host co-evolution as discussed by Lindsay et al. [62], particularly in the context of the life cycles of coccidian parasites and host interactions. 

While pathological lesions associated with protozoal infection of the Eimeriidae family like lymphohistiocytic inflammation or necrosis can be detected in HE-stained sections of infected birds, histologic identification of parasite stages can be very difficult, or they may be overlooked because of their small size [22,57]. In addition, tissue samples of sufficient quality are not always available for light microscopic examination due to advanced autolysis [56]. In such cases, particularly low-grade infections may remain undetected. Conversely, there is a risk of misidentifying cell alterations like karyorrhexis, cellular inclusions, or pigments as merozoites in HE-staining [57]. Therefore, to clearly identify the protozoa in tissue sections and recognize their associated pathogenicity, a CISH procedure for *Isospora* and *Lankesterella* was developed. Furthermore, a semiquantitative scoring system was applied to record parasite burden in the affected organ, and HE-staining was performed to confirm the presence or absence of parasites and to detect associated pathological changes. 

To our knowledge, *Isospora* parasites in birds have not yet been investigated using CISH. Systemic isosporosis with intraleukocytic merozoites in various organs was observed in previously published studies by using HE-staining or tissue impression smears [10,20,63]. In our study, based on visible CISH signals, we were able to detect small parasite stages (presumably merozoites) within mononuclear cells in various organs, and larger gamonts in enterocytes, with the highest parasite burden found in the liver, spleen, and intestine. Especially in organs with a high CISH score, we confirmed the presence of extraintestinal tissue stages (presumably merozoites) in correlating HE-stained sections. This was particularly the case in the liver and spleen. This is consistent with the results of Schrenzel et al. [10], who highlighted the spleen, liver, heart, and lung as frequently affected organs in systemic infections. In numerous other studies, merozoites were detected in the liver or spleen as well [13,20,63,64].

In tissue sections that showed only single signals or small clusters by CISH, parasites could only rarely be identified in corresponding HE-stained sections. One explanation for this result would be that due to the small size of protozoa, the parasite was no longer present in the following HE-stained sections. In addition, autolysis sometimes limited histological evaluation. These results indicate that CISH is a useful tool for identifying *Isospora* stages in tissue sections, even in mild infections or in case the infected tissue is poorly preserved.

For this study, the same set of organs was not always available for examination in each case. For example, bone marrow tissue was only available in a captive *P. ciris* (B1276/12), in which numerous *Isospora* parasites (presumably merozoites) were detected using CISH. This individual suffered from systemic isosporosis and revealed CISH signals in multiple organs, including the liver and spleen. It therefore remains unclear whether this was an isolated case or whether bone marrow is affected more frequently in the case of a systemic infection. Only few studies examined bone marrow for parasites in addition to typically infected organs such as the liver, spleen, and intestine [10,20,54,64]. Although Schrenzel et al. [10] described merozoites in histiocytic or lymphohistiocytic infiltrates of the bone marrow in examined birds, there was no precise information on other affected organs and which bird species was infected. In the past, HE-stained tissue sections from a captive *Spinus tristis* [64] and a *Saltator similis* [20] as well as Giemsa-stained impression smears from wild *P. domesticus* [54] also revealed intraleukocytic parasites (presumably merozoites) in the bone marrow. 

Besides bone marrow, the trachea, esophagus, gonads, crop, and bursa of Fabricius were only available for examination in a few cases, and the occurrence of infected organs based on CISH varied greatly between the cases that included these organs. Some of these organs were also examined for *Isospora* parasites in previous studies [10,22]. Like in the present study, Schrenzel et al. [10] observed histiocytic or lymphohistiocytic infiltrates in the ovaries of passerine birds, in some cases associated with intracellular merozoites. In the work of Sánchez-Cordón et al. [22], the trachea and esophagus were also among the examined organs of three *S. canaria*, all suffering from systemic isosporosis. While parasites were absent in these two organs, one bird showed necrotic foci containing mycotic structures (presumably *Candida* spp.) in its esophagus accompanied by mononuclear cells. Further studies on individuals with a complete set of organs are needed to clarify this question. As far as the authors are aware, this is the first study that examined tissue of the crop and bursa of Fabricius for *Isospora* parasites. 

Scattered signals were visible in blood vessels of various organs, indicating the presence of extraintestinal merozoites, which are transported via circulating leucocytes. These stages can cause a significant mononuclear inflammatory response and necrosis [12,64,65,66,67]. 

It is noteworthy that in four out of five captive birds in which CISH signals were found, at least one organ had a high CISH score, indicating a higher susceptibility of captive birds to severe infection compared to wild birds. This could be explained by the fact that captive animals are generally exposed to higher infection pressure due to the restricted habitat or because they were moved outside their natural range. All captive birds examined in this study appeared to suffer from a systemic isosporosis with lymphocytic to lymphohistiocytic inflammation of multiple organs associated with intralesional parasites, accompanied by severe liver necrosis in two cases. In three of these captive birds, the involved lineages were only found in these species. However, in the fourth case (*C. carduelis*, B421/13), the same lineage occurred in a wild *S. atricapilla*.

*Isospora*-associated tissue lesions were consistent with the results of previous studies [19,20,21,22,63]. In many cases of our study, a lymphocytic to lymphohistiocytic inflammation of varying severity was noted in various organs, sometimes accompanied by necrosis in the liver and spleen. The severity of histologic lesions often appeared to correlate with the CISH score. Unfortunately, it was difficult to describe the detailed morphological appearance of *Isospora* stages in HE-stained tissue sections. Intracytoplasmic merozoites were often only faintly stained and could sometimes hardly be distinguished from cell structures of the host tissue cell due to autolytic changes. Partington et al. [57] also described the identification of parasites in HE-stained tissue samples as challenging, except for severe cases. In the past, merozoites of *Isospora* have been detected and morphologically described in mononuclear cells using impression smears of the liver, spleen, lung, kidney, and intestine [15,20]. Impression smears could therefore serve as an additional method for morphological description. However, this method has been described as unreliable for low-grade, subclinical infections [19].

Previous knowledge about the life cycle of *Isospora* (Figure 1) is consistent with our findings of intracytoplasmic merozoites in mononuclear cells such as lymphocytes and macrophages in various organs and gamonts in the intestinal epithelium. It is noteworthy that every bird with detectable CISH signals in the intestine also showed signals in at least one other organ (in most cases the liver). This indicates that all CISH-positive birds suffered from a systemic infection and raises the question of whether there are really *Isospora* infections with only an intestinal life cycle. Further investigations are required to answer this question. 

These observations justify the rationale of the present study to use the data of blood or tissue investigations as a basis for judging whether a case is *Isospora*-infected or not. The number of *Isospora*-positive cases might have been higher by consistently examining intestinal samples or feces (as suggested by the detection of single *Isospora*-cases in PCR-negative samples), but fecal samples were not available in the current study. Kubiski et al. [24] followed a different approach by a priori selecting samples from birds with diagnosed protozoal infections (*Isospora*, Coccidia), which were mostly collected by the San Diego Zoo Wildlife Alliance. They generated sequences from 136 individual birds, whereby intestines or feces were only available for half of the individuals. They also detected *Isospora* DNA in liver, lung, and spleen samples originating from the other half of bird specimens, supporting the presence of parasite stages in the blood at least. Illera et al. [58] screened fresh fecal samples of 248 *Curruca conspicillata* from Macaronesia, Morocco, and Spain and detected *Isospora* spp. at all 14 sites studied, with infection rates ranging from 7.7% to 52.0%. The infection rates for *Isospora* spp. determined from fecal samples were thus much higher than those in blood samples of Sylviidae screened for the present study with only 2.5% for *S. atricapilla*. Thus, the analysis of blood and non-intestinal tissue samples likely led to much lower infection rates detected than actually present in these birds. 

To the best of our knowledge, the present study is the first to detect the presence of *Lankesterella* in tissue sections of passerine birds using CISH. The percentage of samples showing CISH signals to confirm the presence of parasites was lower in *Lankesterella*-PCR-positive birds (60%) than in *Isospora*-PCR-positive birds (85%). CISH signals of *Lankesterella* parasite stages were found more frequently in the spleen, lung, and brain, followed by the liver, kidney, and heart. Unfortunately, as with *Isospora*, the same set of organ samples was not available for each case. One explanation for the lower presence in CISH compared to *Isospora* could be that the spleen, which appeared most frequently affected, was missing in some CISH-negative birds. All *Lankesterella*-infected organs showed only a low parasite burden with predominantly scattered signals. HE-staining was subsequently performed to visualize parasite stages identified by CISH in tissue sections. However, parasitic stages could not be identified with certainty in corresponding HE-stained sections in any case. Interestingly, signals were frequently found closely associated with the endothelia of different-sized blood vessels in various organs such as the lungs, heart, liver, and brain. This correlates with the results of previously published studies on *Lankesterella* species in anurans, where developmental stages such as meronts and/or oocysts were detected in capillary endothelial cells and in macrophages of the liver, spleen, lung, or kidney [30,33]. Furthermore, zygotes and oocysts of *Lankesterella* have been found in endothelial cells of blood vessels in the muscle, intestinal wall, and ovary and in the envelopes of the brain of *B. marinus* [32]. Desser et al. [30] proposed vascular endothelial cells as the primary site for oocyst formation, which, when sporulated, can contain 32 or more sporozoites lacking a sporocyst [31]. In anurans, sporozoites released from oocysts were observed in hepatocytes and macrophages of the liver parenchyma before being established in circulating erythrocytes [31,33,34]. 

In case the life cycle of *Lankesterella* in birds is similar to that of amphibians, observed CISH signals closely associated with the endothelia could therefore represent meronts or oocytes. This demonstrates the diagnostic value of CISH as a method for detecting *Lankesterella* stages in tissue sections, providing further information on the life cycle of *Lankesterella* in passerines. In addition, molecular techniques such as CISH are an important method for distinguishing between parasites of the order Eucoccidiorida such as *Lankesterella*, *Isospora*, and *Hepatozoon*, which were often confused in the past due to morphologically similar life stages [3,9].

Although studies that have investigated *Lankesterella* species in amphibians provide insights into the developmental stages of the parasites [29,30,32,33,34,68], the pathogenicity of *Lankesterella* in anurans has hardly been investigated so far. And if so, it remained unclear to what extent the protozoan infection contributed to the host’s death [31]. 

Corresponding pathological changes associated with CISH signals for *Lankesterella* were absent in the examined birds. Although some birds showed predominantly mild inflammation in various organs, these were not associated with CISH signals. Therefore, *Lankesterella* parasites were not considered to be responsible for the observed lesions, and the actual etiology of the detected pathological changes remained unclear. These results indicate subclinical infection and imply that the individuals may have served merely as reservoir hosts. 

## 5. Conclusions

In the present study, infection with *Isospora* and *Lankesterella* was detected in 64 species of wild passerine birds using PCR and sequencing. The overall infection rate of *Lankesterella* was 10.7% and therefore slightly higher than the infection rate of *Isospora* which was 5.8%. Partial *CytB* sequence analysis revealed 26 *Isospora* and 25 *Lankesterella* lineages, with the majority of lineages detected only in one bird species, indicating high host specificity. Using the designed specific probes, parasite stages of *Isospora* and *Lankesterella* could be detected and differentiated in tissue sections of infected birds by CISH. Systemic isosporosis, formerly described as atoxoplasmosis, resulted in some cases in significant pathological lesions in the hosts’ organs. We therefore postulate that wild birds are not only subclinically infected with *Isospora* but can also become severely ill. By contrast, low detection of CISH signals for *Lankesterella* in tissue sections and the absence of corresponding pathological changes in HE-stained sections indicate more frequent subclinical infections in wild passerines.

## Figures and Tables

**Table 1:** Bird species investigated for *Isospora* and *Lankesterella* parasites. For each host species, infection rates (IRs) of *Isospora* and *Lankesterella* parasites and detected *cytochrome b* (*CytB*) lineages are indicated with numbers of records in brackets. Note that some individuals had mixed infections with two or three lineages of the same parasite genus.

Host Family	Host Species	N	IR (%)	*Isospora* Lineage	IR (%)	*Lankesterella* Lineage
Acrocephalidae	*Acrocephalus arundinaceus*	3	-		-	
	*Acrocephalus melanopogon*	9	-		11.1	kACRSCH01 (1)
	*Acrocephalus palustris*	19	5.3	iACRPAL01 (1)	10.5	kACRPAL02 (2)
	*Acrocephalus schoenobaenus*	8	-		37.5	kACRSCH01 (2), kACRSCH02 (2)
	*Acrocephalus scirpaceus*	22	9.1	iACRSCI01 (1), iACRSCI02 (1), iACRPAL01 (1)	-	
	*Acrocephalus* sp.	1	-		-	
	*Hippolais icterina*	1	-		-	
Aegithalidae	*Aegithalos caudatus*	1	-		-	
Alaudidae	*Calandrella brachydactyla*	1	-		-	
Certhiidae	*Certhia brachydactyla*	1	-		-	
Corvidae	*Garrulus glandarius*	5	-		-	
Emberizidae	*Emberiza citrinella*	10	20.0	iEMBCIR01 (1), iEMBCIR02 (1)	-	
	*Emberiza schoeniclus*	7	-		-	
Fringillidae	*Carduelis carduelisF*	3	-		-	
	*Coccothraustes coccothraustes*	19	-		-	
	*Fringilla coelebs*	6	16.6	iFRICOE01 (1)	-	
	*Pyrrhula pyrrhula*	7	-		-	
Hirundinidae	*Delichon urbicum*	3	-		33.3	kHIRRUS01 (1)
	*Hirundinidae* sp.	1	-		-	
	*Hirundo rustica*	43	-		14.0	kHIRRUS01 (4), kSYLATR01 (2)
	*Hirundo* sp.	1	-		-	
Laniidae	*Lanius collurio*	7	-		42.9	kLANCOL01 (2), kLANCOL02 (1)
Locustellidae	*Locustella luscinioides*	9	-		-	
	*Locustella naevia*	1	-		-	
Motacillidae	*Motacilla alba*	4	-		50.0	kMOTALB01 (2)
	*Motacilla flava*	1	-		-	
Muscicapidae	*Erithacus rubecula*	104	5.8	iERIRUB01 (1), iERIRUB02 (3), iERIRUB03 (1), iERIRUB04 (1)	21.2	kERIRUB01 (21), kERIRUB02 (1), kSYLATR01 (1)
	*Ficedula hypoleuca*	11	-		45.5	kFICHYP01 (4), kFICHYP02 (1)
	*Luscinia luscinia*	1	100.0	iLUSLUS01 (1)	-	
	*Luscinia megarynchos*	5	-		-	
	*Muscicapa striata*	1	-		-	
	*Phoenicurus ochruros*	8	62.5	iPHOOCH01 (4), iPHOOCH02 (1)	-	
	*Phoenicurus phoenicurus*	3	-		-	
	*Phoenicurus* sp.	1	-		-	
	*Saxicola rubicola*	1	-		-	
Panuridae	*Panurus biarmicus*	4	-		-	
Paridae	*Cyanistes caeruleus*	65	-		1.5	kCYACAE01 (1)
	*Parus major*	86	3.5	iPARMAJ01 (1), iPASDOM01 (1), iPASDOM02 (1)	7.0	kPARMAJ01 (4), kPARMAJ02 (2)
	*Parus* sp.	1	-		-	
	*Periparus ater*	1	-		-	
	*Poecile palustris*	8	-		-	
Passeridae	*Passer domesticus*	46	50.0	iPASDOM01 (5), iPASDOM02 (7), iPASDOM03 (8), iPASDOM04 (1), iPASDOM05 (1), iPASDOM06 (1)	6.5	kPASDOM01 (1), kPASDOM03 (1), kPASDOM04 (1)
	*Passer montanus*	8	12.5	iPASMON01 (1)	-	
	*Passer* sp.	3	100.0	iPASDOM01 (1), iPASDOM02 (2)	33.3	kPASDOM02 (1)
Phylloscopidae	*Phylloscopus collybita*	18	11.1	iPHYCOL01 (2)	5.6	kPHYCOL01 (1)
	*Phylloscopus sibilatrix*	4	-		-	
	*Phylloscopus trochiloides*	2	-		-	
	*Phylloscopus trochilus*	18	16.7	iPHYCOL01 (2), iPHYTRO01 (1)	5.6	kPHYCOL02 (1)
Prunellidae	*Prunella modularis*	14	-		21.4	kPRUMOD01 (3)
Regulidae	*Regulus ignicapilla*	3			-	
	*Regulus regulus*	11	27.3	iREGREG01 (2), iREGREG02 (1)	9.1	kREGREG01 (1)
Sylviidae	*Sylvia atricapilla*	118	2.5	iSYLATR01 (1), iSYLATR02 (2)	17.8	kSYLATR01 (18), kSYLATR02 (1), kACRSCH01 (1), kPARMAJ01 (1)
	*Sylvia borin*	7	-		-	
	*Curruca communis*	9	-		11.1	kSYLCOM01 (1)
	*Curruca curruca*	10	-		20.0	kSYLCUR01 (2)
	*Sylvia* sp.	1	-		-	
Troglodytidae	*Troglodytes troglodytes*	5	-		-	
Turdidae	*Turdus merula*	28	-		7.1	kTURMER01 (1), kSYLATR01 (1)
	*Turdus philomelos*	12	-		-	
Captive birds						
Cardinalidae	*Passerina ciris*	1	100.0	iPASCIR01 (1)	-	
Estrildidae	*Padda oryzivora*	1	100.0	iPADORY01 (1)	-	
Fringillidae	*Carduelis carduelis*	1	100.0	iSYLATR01 (1)	-	
	*Spinus cucullatus*	3	-		-	
	*Serinus canaria*	3	33.3	iSERCAN01 (1)	-	
	*Spinus xanthogastrus*	3	-		-	
	*Spinus yarrellii*	3	33.3	iSPIYAR01 (1)	-	

**Table 2:** Results of chromogenic in situ hybridization (CISH) targeting *Isospora* parasites in bird tissue.

Host Family	Host Species	Host-ID	CISH	HE	LU	LI	SP	KI	BR	MU	GI	IN	TR	ES	TE	OV	BU	PC	CR	BM
Acrocephalidae	*Acrocephalus palustris*	AH1899	pos	+	n/a	+	n/a	+	−	−	n/a	n/a	n/a	n/a	n/a	n/a	n/a	n/a	n/a	n/a
	*Acrocephalus scirpaceus*	AH1896	pos	+	+	+	+	−	−	−	n/a	n/a	n/a	n/a	n/a	n/a	n/a	n/a	n/a	n/a
Cardinalidae	*Passerina ciris* (c)	B1276/12	pos	+	++	+++	+++	++	+	+	++	+	n/a	n/a	n/a	++	n/a	n/a	++	+++
Estrildidae	*Padda oryzivora* (c)	B727/17	pos	+	+	+++	+++	+	+	+	++	+++	n/a	n/a	n/a	n/a	n/a	+	n/a	n/a
Fringillidae	*Carduelis carduelis*	B421/13	pos	n/a	n/a	+++	n/a	+	n/a	n/a	n/a	n/a	n/a	n/a	n/a	n/a	n/a	n/a	n/a	n/a
	*Serinus canaria* (c)	B1277/12	neg	n/a	n/a	n/a	n/a	n/a	−	−	−	n/a	n/a	n/a	n/a	n/a	n/a	n/a	n/a	n/a
	*Spinus yarrellii* (c)	T331/12	pos	−	−	++	+++	+	−	n/a	+	+	n/a	n/a	n/a	n/a	n/a	+	n/a	n/a
Muscicapidae	*Erithacus rubecula*	AH1980	pos	−	−	+	+	−	−	−	−	+++	n/a	n/a	n/a	−	n/a	−	n/a	n/a
	*Erithacus rubecula*	AH2000	neg	−	−	−	n/a	−	−	−	−	−	n/a	n/a	−	n/a	n/a	n/a	n/a	n/a
	*Phoenicurus ochruros*	AH0792	pos	−	−	+	+	n/a	−	n/a	−	−	−	−	n/a	n/a	n/a	−	n/a	n/a
	*Phoenicurus ochruros*	AH1977	pos	−	n/a	+	n/a	−	−	−	−	+	n/a	n/a	n/a	n/a	n/a	−	n/a	n/a
	*Phoenicurus ochruros*	AH1988	pos	−	−	−	n/a	+	−	−	−	+	n/a	−	n/a	n/a	n/a	n/a	n/a	n/a
	*Phoenicurus ochruros*	AH2009	pos	−	−	+	n/a	−	−	−	−	+	−	−	−	n/a	n/a	n/a	n/a	n/a
Paridae	*Parus major*	AH1998	neg	−	−	−	−	−	−	−	−	−	n/a	n/a	n/a	n/a	−	n/a	n/a	n/a
	*Parus major*	AH1999	pos	−	−	+	n/a	−	−	−	−	++	n/a	n/a	n/a	−	n/a	n/a	n/a	n/a
Passeridae	*Passer domesticus*	AH0597	pos	−	−	n/a	+++	−	−	n/a	++	n/a	n/a	n/a	n/a	n/a	n/a	n/a	n/a	n/a
	*Passer domesticus*	AH1553	neg	−	−	−	−	−	−	n/a	n/a	n/a	n/a	n/a	−	n/a	n/a	n/a	n/a	n/a
	*Passer domesticus*	AH1557	pos	−	n/a	−	+	n/a	−	n/a	n/a	n/a	n/a	n/a	n/a	n/a	n/a	n/a	n/a	n/a
	*Passer domesticus*	AH1559	pos	+	−	+	+	+	−	n/a	n/a	n/a	n/a	n/a	n/a	n/a	n/a	n/a	n/a	n/a
	*Passer domesticus*	AH1562	pos	−	+	+	+++	++	−	n/a	++	n/a	n/a	+	n/a	n/a	n/a	n/a	n/a	n/a
	*Passer domesticus*	AH1566	pos	−	+	++	+++	+	−	n/a	n/a	n/a	n/a	n/a	n/a	+	n/a	n/a	n/a	n/a
	*Passer domesticus*	AH1570	pos	−	n/a	++	n/a	+	−	n/a	n/a	n/a	n/a	n/a	n/a	n/a	n/a	n/a	n/a	n/a
	*Passer domesticus*	AH2031	pos	+	−	−	n/a	−	−	+	+	+	−	−	−	n/a	n/a	n/a	n/a	n/a
	*Passer montanus*	AH0761	pos	−	+	+	+	n/a	+	n/a	n/a	n/a	n/a	n/a	n/a	n/a	n/a	n/a	n/a	n/a
	*Passer* sp.	AH1989	pos	−	+	−	+	−	−	+	+	+	n/a	−	n/a	n/a	−	+	n/a	n/a
	*Passer* sp.	AH2026	pos	+	+	+	n/a	−	−	+	−	+	−	−	−	n/a	n/a	n/a	n/a	n/a

Semiquantitative CISH results for *Isospora* of PCR-positive wild and captive (c) birds scored as absence of signals (−), low-grade (+), moderate (++), or high-grade (+++) in context with the location of signals within the heart (HE), lung (LU), liver (LI), spleen (SP), kidney (KI), brain (BR), skeletal muscle (MU), gizzard and/or proventriculus (GI), intestine (IN), trachea (TR), esophagus (ES), testicle (TE), ovary (OV), bursa of Fabricius (BU), pancreas (PC), crop (CR), and bone marrow (BM). n/a: organ not available; pos: positive (bird showed CISH signals in at least one organ); neg: negative (no signal in organs detected).

**Table 3:** Results of CISH targeting *Lankesterella* parasites in bird tissue.

Host Family	Host Species	Host-ID	CISH	HE	LU	LI	SP	KI	BR	MU	GI	IN	TR	ES	TE	OV	BU	PC	CR	BM
Acrocephalidae	*Acrocephalus melanopogon*	AH1591	pos	n/a	n/a	n/a	n/a	n/a	+	n/a	n/a	n/a	n/a	n/a	n/a	n/a	n/a	n/a	n/a	n/a
Emberizidae	*Emberiza citrinella*	AH1888	neg	−	−	−	−	−	−	−	n/a	n/a	n/a	n/a	n/a	n/a	n/a	n/a	n/a	n/a
Hirundinidae	*Hirundo rustica*	AH2003	pos	−	−	−	n/a	−	+	−	−	n/a	−	−	n/a	n/a	−	n/a	n/a	n/a
Motacillidae	*Motacilla alba*	AH1897	pos	+	+	+	+	+	+	+	n/a	n/a	n/a	n/a	+	n/a	n/a	−	n/a	n/a
	*Motacilla alba*	AH1969	pos	+	n/a	+	+	+	+	−	+	+	n/a	n/a	+	n/a	n/a	−	n/a	n/a
Muscicapidae	*Erithacus rubecula*	AH1580	neg	n/a	n/a	n/a	n/a	n/a	−	n/a	n/a	n/a	n/a	n/a	n/a	n/a	n/a	n/a	n/a	n/a
	*Erithacus rubecula*	AH1634	pos	n/a	n/a	n/a	n/a	n/a	+	n/a	n/a	n/a	n/a	n/a	n/a	n/a	n/a	n/a	n/a	n/a
	*Erithacus rubecula*	AH1966	pos	−	+	+	n/a	−	−	−	−	−	n/a	−	n/a	−	n/a	−	n/a	n/a
	*Erithacus rubecula*	AH1970	neg	−	n/a	−	n/a	−	−	−	−	−	n/a	−	n/a	n/a	n/a	−	n/a	n/a
	*Erithacus rubecula*	AH2139	neg	−	−	−	n/a	−	−	−	−	−	−	−	n/a	n/a	n/a	n/a	n/a	n/a
	*Erithacus rubecula*	AH2148	pos	−	n/a	n/a	n/a	n/a	n/a	+	−	n/a	n/a	n/a	n/a	n/a	n/a	n/a	n/a	n/a
Paridae	*Cyanistes caeruleus*	AH2043	neg	n/a	−	−	−	−	−	n/a	n/a	n/a	n/a	n/a	n/a	n/a	n/a	n/a	n/a	n/a
	*Parus major*	AH2039	neg	−	−	−	n/a	−	−	−	−	−	−	−	n/a	n/a	n/a	n/a	n/a	n/a
Passeridae	*Passer domesticus*	AH1569	neg	−	−	−	−	−	−	n/a	n/a	n/a	n/a	n/a	n/a	n/a	n/a	n/a	n/a	n/a
	*Passer domesticus*	AH0462	pos	−	−	−	n/a	−	−	n/a	+	−	n/a	n/a	n/a	n/a	n/a	n/a	n/a	n/a
Regulidae	*Regulus regulus*	AH2146	neg	−	−	−	n/a	−	−	−	−	−	−	−	n/a	n/a	n/a	n/a	n/a	n/a
Sylviidae	*Sylvia atricapilla*	AH1894	pos	−	−	−	+	−	−	−	n/a	n/a	n/a	n/a	n/a	n/a	n/a	n/a	n/a	n/a
	*Sylvia atricapilla*	AH1905	pos	−	+	−	−	−	−	−	n/a	n/a	n/a	n/a	−	n/a	n/a	n/a	n/a	n/a
	*Sylvia atricapilla*	AH1991	pos	+	+	−	+	+	−	−	−	n/a	n/a	n/a	−	n/a	−	n/a	n/a	n/a
	*Curruca communis*	AH1900	pos	−	+	−	−	−	+	−	n/a	n/a	n/a	n/a	−	n/a	n/a	n/a	n/a	n/a

Semiquantitative CISH results for *Lankesterella* of PCR-positive wild birds scored as absence of signals (−), low-grade (+), moderate (++), or high-grade (+++) in context with the location of signals within the heart (HE), lung (LU), liver (LI), spleen (SP), kidney (KI), brain (BR), skeletal muscle (MU), gizzard and/or proventriculus (GI), intestine (IN), trachea (TR), esophagus (ES), testicle (TE), ovary (OV), bursa of Fabricius (BU), pancreas (PC), crop (CR), and bone marrow (BM). n/a: organ not available; pos: positive (bird showed CISH signals in at least one organ); neg: negative (no signal in organs detected).

## Data Availability

Data are contained within the article. The generated sequence data were deposited in the NCBI GenBank database.

## Supplementary Materials

The following supporting information can be downloaded at https://www.mdpi.com/article/10.3390/pathogens13040337/s1: Figure S1: Histological sections of the performed probe cross-checking using chromogenic in situ hybridization (A–D). Intestine of a wild *E. rubecula* (AH1980), which was tested positive for *Isospora* by PCR and sequencing, showed abundant *Isospora*-specific signals within the enterocytes (A), whereas no *Lankesterella*-specific signals were present (B). A *Lankesterella*-specific signal (D) was detected in the heart of an infected *M. alba* (AH1969, *Lankesterella* infection was confirmed by PCR and sequencing), but *Isospora*-specific signals were absent (C). This confirms the specificity of the probes and rules out cross-reactivity. Scale bars: 50 µm; Figure S2: Bayesian Inference tree of partial *cytochrome c oxidase I* sequences (648 bp) of avian *Isospora* parasites from GenBank and the present study. Posterior probabilities and maximum likelihood bootstrap values are indicated at most nodes. The scale bar indicates the expected mean number of substitutions per site according to the model of sequence evolution applied. A sequence of *Lankesterella* sp. kERIRUB01 from *E. rubecula* was included as outgroup; Figure S3: Bayesian Inference trees of partial concatenated *COI* (860 bp) and *CytB* (827 bp) sequences of *Isospora* (A) and *Lankesterella* (B) lineages. Posterior probabilities and maximum likelihood bootstrap values are indicated at all nodes. The scale bars indicate the expected mean number of substitutions per site according to the model of sequence evolution applied. Sequence of *Lankesterella* sp. kSYLATR01 and *Isospora* sp. iPASDOM02 were included as outgroups; Figure S4: Histological sections of the proventriculus (A–D) and brain (I–L) of a captive *P. ciris* (B1276/12) and serosa of the proventriculus (E–H) of a wild *P. domesticus* (AH0597). The images in the right-hand column show higher magnifications of the regions marked by rectangles in the left columns. In the HE-stained sections, the proventriculus showed lymphocytic to lymphohistiocytic infiltrates with intracytoplasmic *Isospora* parasites (C,G, white arrowheads, presumably merozoites). Note the *Isospora* parasites (white arrowhead, presumably merozoites) inside the wall of a blood vessel in the brain (K). Due to their small size, the parasites were not always readily identifiable in HE-stained sections but could easily be detected using CISH (B,D,F,H,J,L). Scale bars: (A,B,E,F,I,J) 50 µm; (C,D,G,H,K,L) 10 µm; Figure S5: Histological sections of the intestine (A–C) of a wild *E. rubecula* (AH1966), which was confirmed to be infected with *Lankesterella* by PCR and sequencing and showed *Lankesterella*-specific chromogenic in situ hybridization signals in liver and lung. Parasite stages (white arrow) were also found in the intestinal epithelium (A), but *Lankesterella*-specific CISH signals were not seen in the tissue (B). Cross-testing with the *Isospora*-specific probe revealed a positive staining, indicating a co-infection (C). Scale bars: 50 µm; Table S1: Genetic p-distances between the *Isospora cytochrome b* lineages (823 bp) detected in the present study. A sequence of *Lankesterella* sp. kPASDOM02 was added for comparison; Table S2: Genetic p-distances between the *Lankesterella cytochrome b* lineages (823 bp) detected in the present study. A sequence of *Isospora* sp. iSYLATR01 was added for comparison.
