# Supplementary material for: Isospora and Lankesterella Parasites (Eimeriidae, Apicomplexa) of Passeriform Birds in Europe: Infection Rates, Phylogeny, and Pathogenicity
Source: Pathogens. 2024 Apr 18;13(4):337. doi: 10.3390/pathogens13040337 (PMC11053544; doi:10.3390/pathogens13040337)
Supplement: Supplementary file 1 [file pathogens-13-00337-s001.zip › Supplementary file Figure S2.pdf]

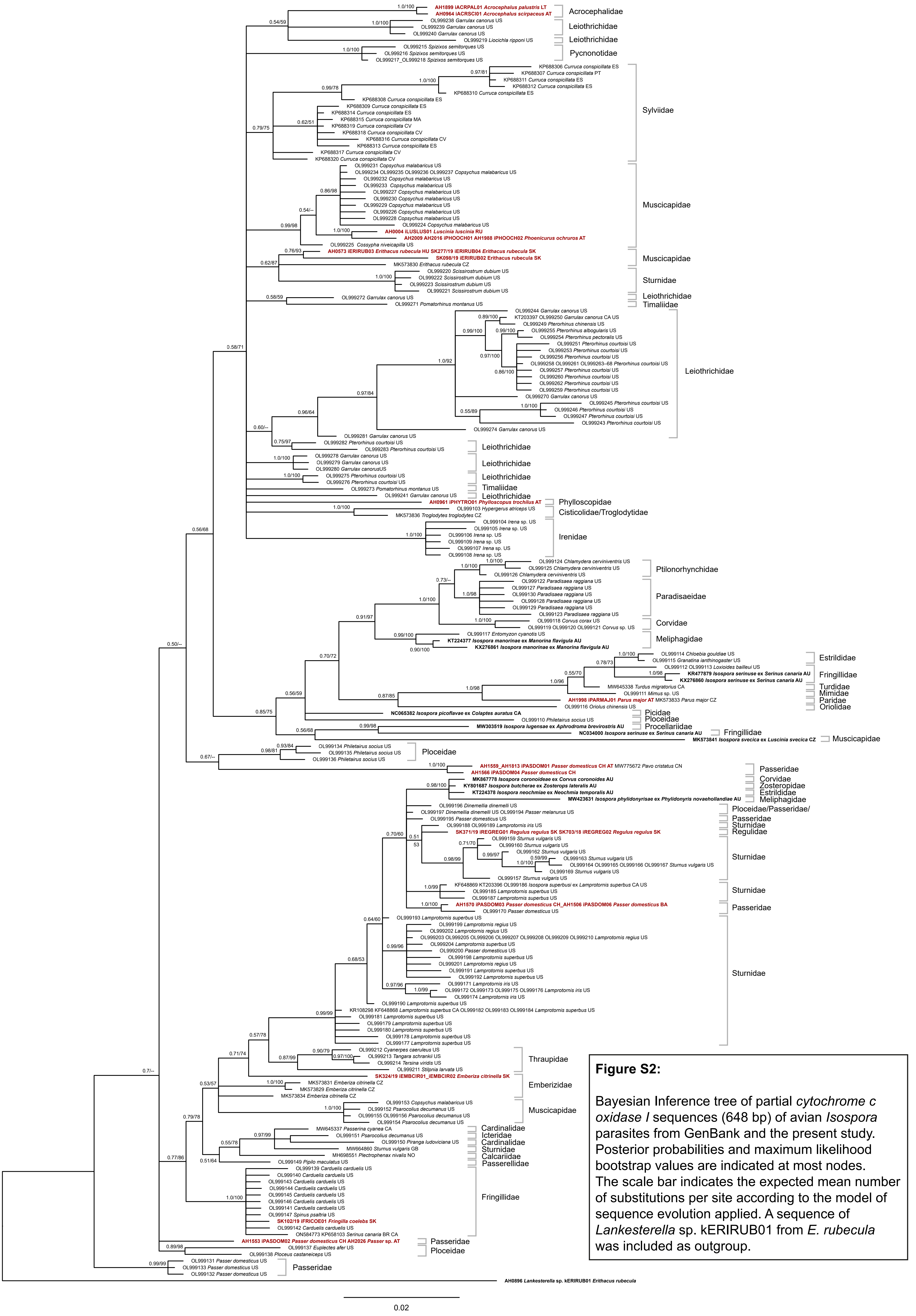

Figure S2:

Bayesian Inference tree of partial *cytochrome c oxidase I* sequences (648 bp) of avian *Isospora* parasites from GenBank and the present study. Posterior probabilities and maximum likelihood bootstrap values are indicated at most nodes. The scale bar indicates the expected mean number of substitutions per site according to the model of sequence evolution applied. A sequence of *Lankesterella* sp. kERIRUB01 from *E. rubecula* was included as outgroup.
