# Supplementary material for: Isospora and Lankesterella Parasites (Eimeriidae, Apicomplexa) of Passeriform Birds in Europe: Infection Rates, Phylogeny, and Pathogenicity
Source: Pathogens. 2024 Apr 18;13(4):337. doi: 10.3390/pathogens13040337 (PMC11053544; doi:10.3390/pathogens13040337)
Supplement: Supplementary file 1 [file pathogens-13-00337-s001.zip › Supplementary file Figure S3.pdf]

Figure S3

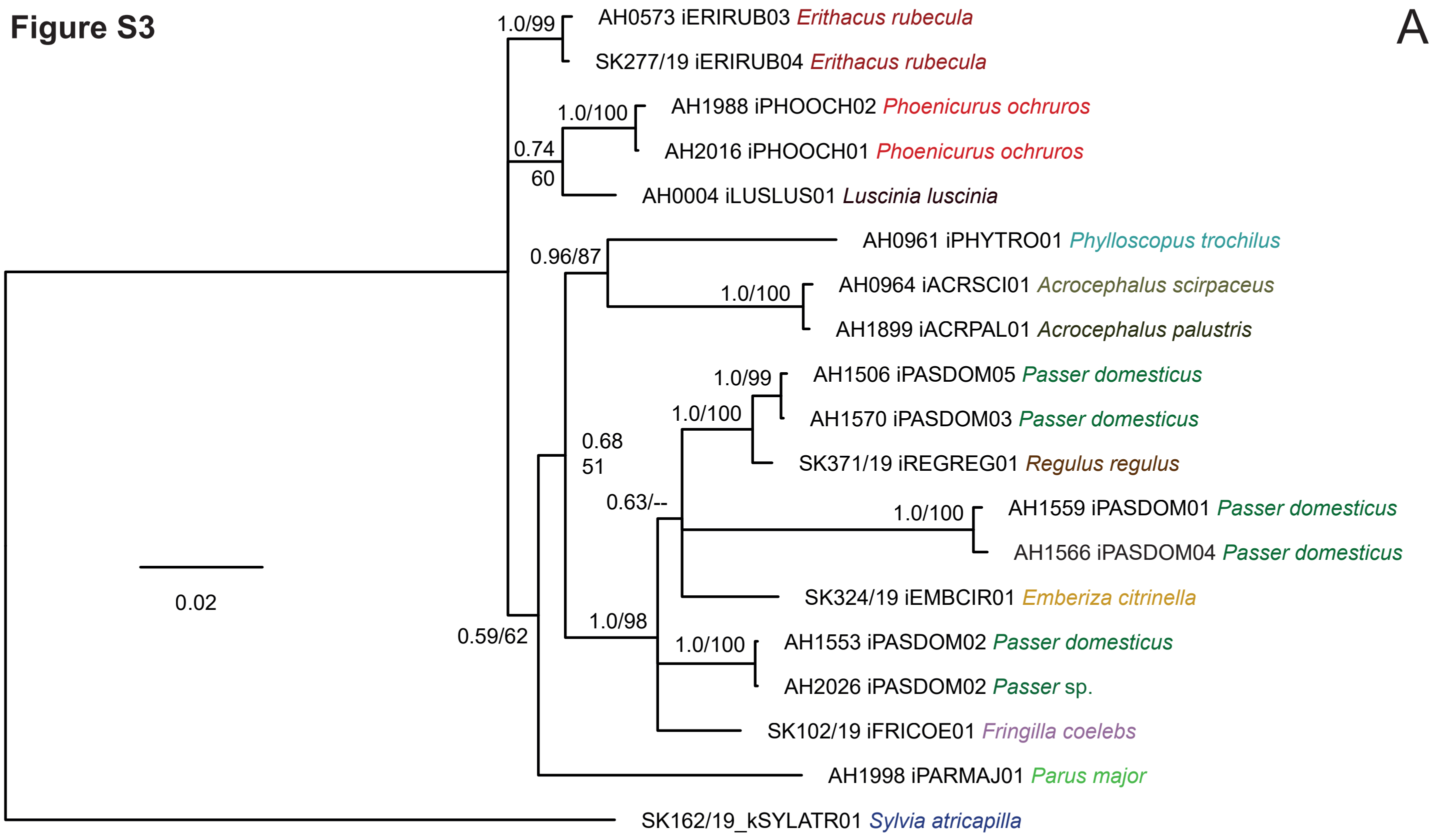

Bayesian Inference tree of partial concatenated *Isospora COI* (860 bp) and *CytB* (827 bp) sequences. Posterior probabilities and maximum likelihood bootstrap values are indicated at all nodes. The scale bar indicates the expected mean number of substitutions per site according to the model of sequence evolution applied. A sequence of *Lankesterella* sp. kSYLATR01 was included as outgroup.

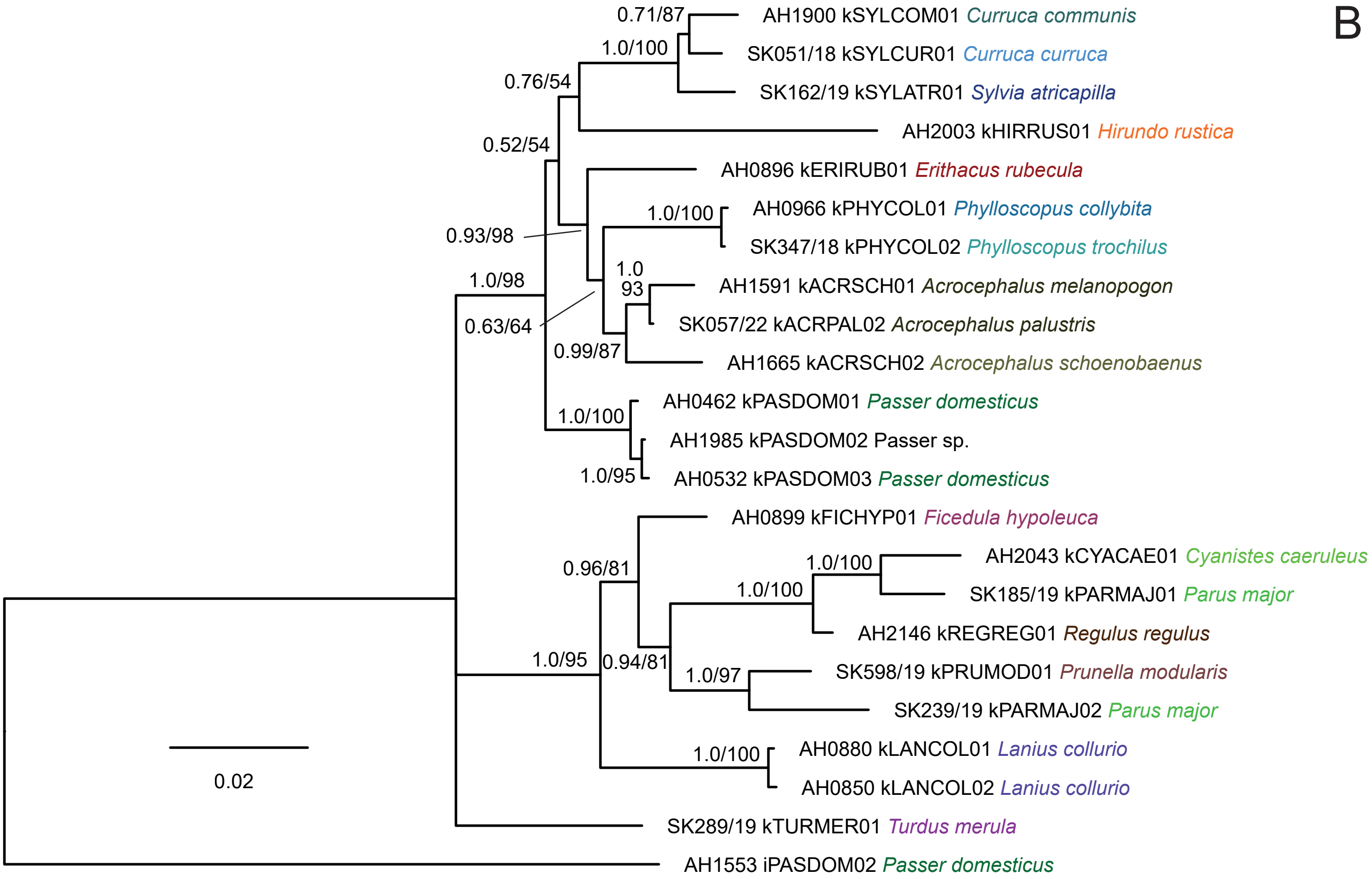

Bayesian Inference tree of partial concatenated *Lankesterella COI* (860 bp) and *CytB* (827 bp) sequences. Posterior probabilities and maximum likelihood bootstrap values are indicated at all nodes. The scale bar indicates the expected mean number of substitutions per site according to the model of sequence evolution applied. A sequence of *Isospora* sp. iPASDOM02 was included as outgroup.
