# Supplementary figures and images for: Isospora and Lankesterella Parasites (Eimeriidae, Apicomplexa) of Passeriform Birds in Europe: Infection Rates, Phylogeny, and Pathogenicity
Source: Pathogens. 2024 Apr 18;13(4):337. doi: 10.3390/pathogens13040337 (PMC11053544; doi:10.3390/pathogens13040337)

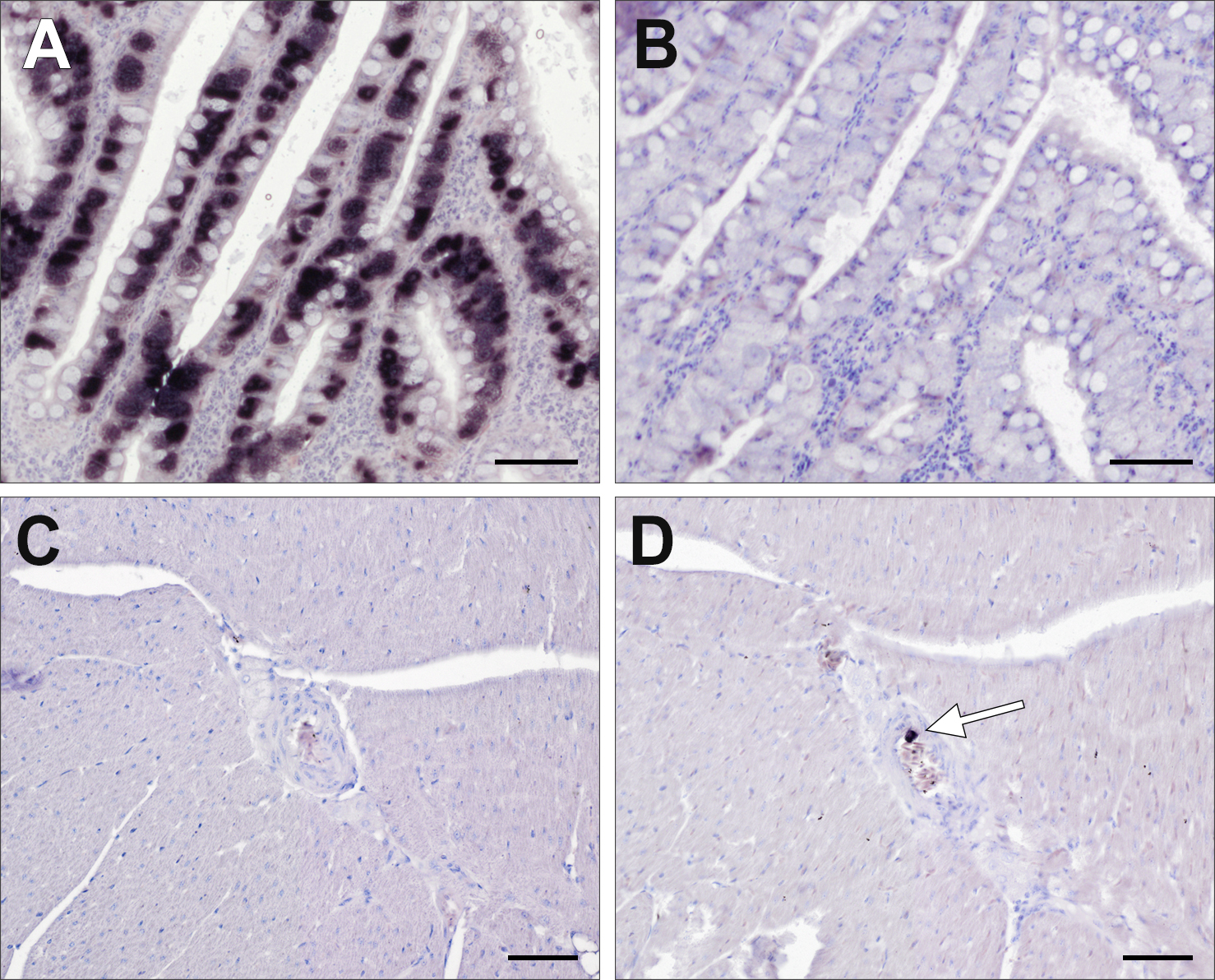

Supplement: Supplementary file 1 [file pathogens-13-00337-s001.zip › Supplementary file Figure S1.jpg]

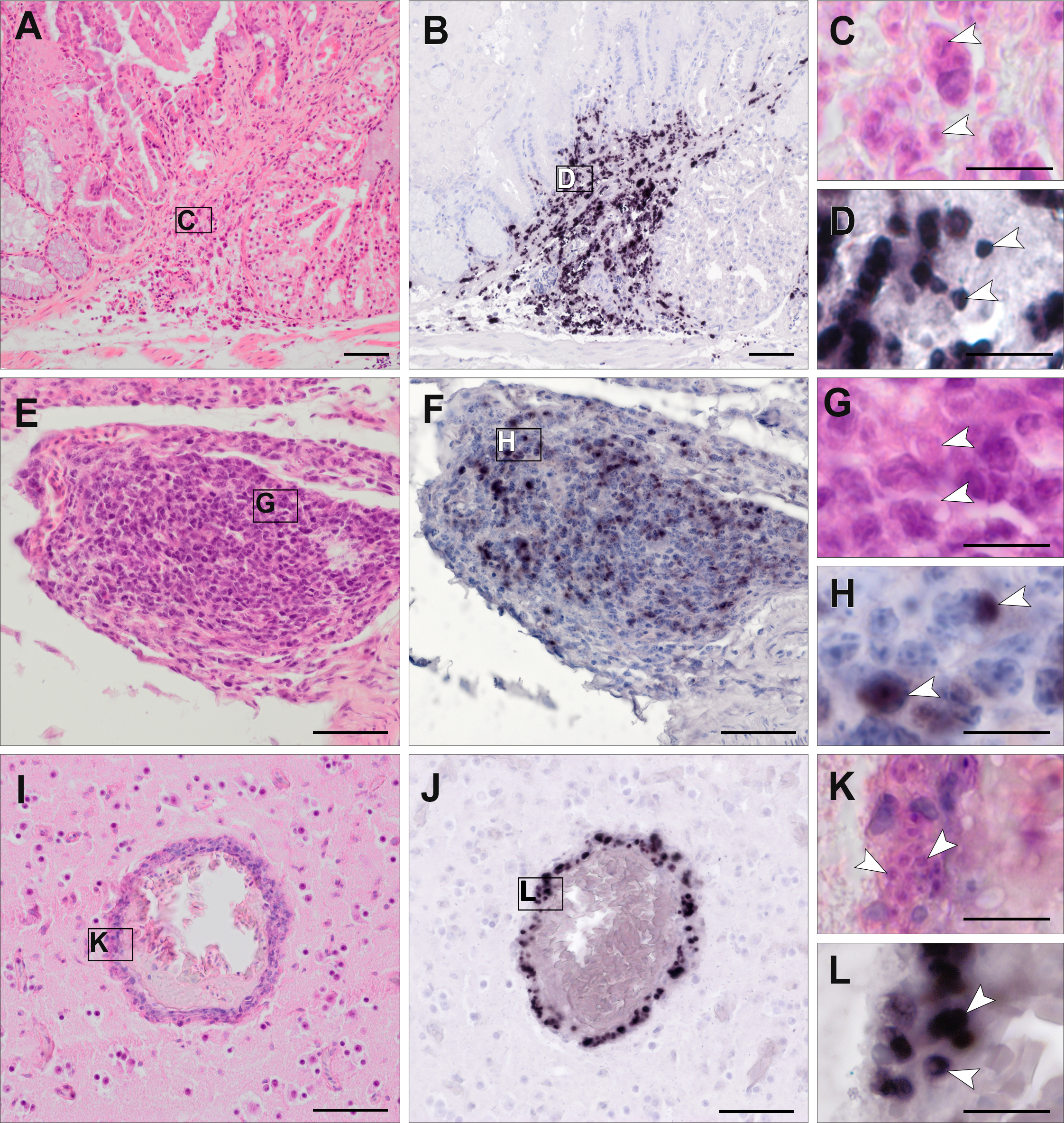

Supplement: Supplementary file 1 [file pathogens-13-00337-s001.zip › Supplementary file Figure S4.jpg]

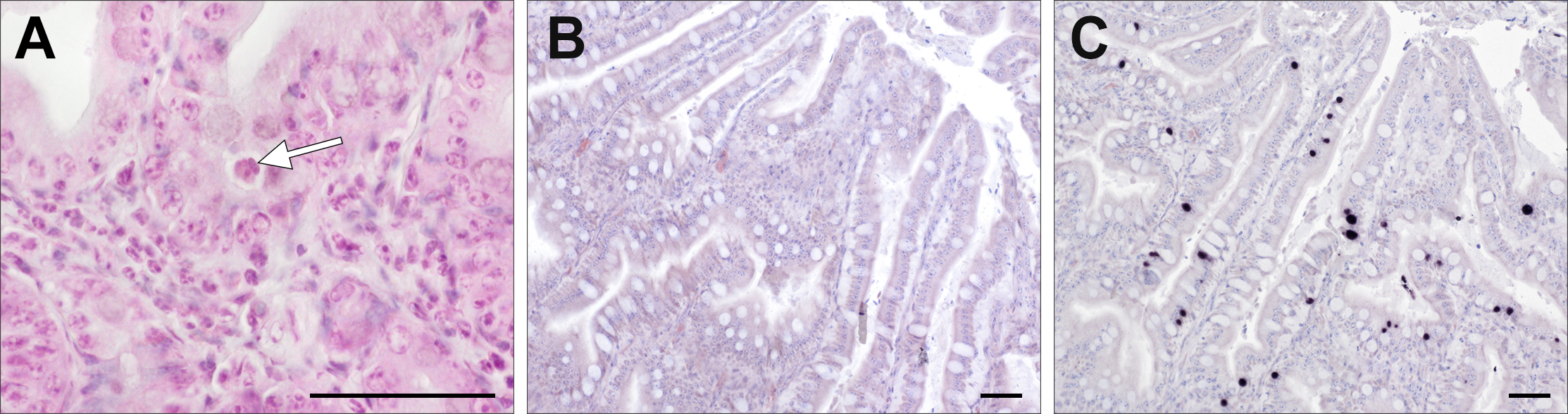

Supplement: Supplementary file 1 [file pathogens-13-00337-s001.zip › Supplementary File Figure S5.jpg]
